# Supplementary material for: Bi-allelic variants in WDR47 cause a complex neurodevelopmental syndrome
Source: EMBO Mol Med. 2024 Nov 28;17(1):129–68. doi: 10.1038/s44321-024-00178-z (PMC11730659; doi:10.1038/s44321-024-00178-z)
Supplement: Supplementary file 2 — Appendix file [file 44321_2024_178_MOESM2_ESM.pdf]

Appendix

Bi-allelic variants in *WDR47* cause a complex neurodevelopmental syndrome

Bayam et al.

Content:

- **Appendix Supplementary information**: Clinical features of patients with *WDR47* variants, related to Figure 1. .... Page 2
- **Appendix Figure S1**: Patients with *WDR47* variants, related to Figure 2. .... Page 6
- **Appendix Figure S2**: Validation of the mouse models used in the study, related to Figure 3. Page 7
- **Appendix Figure S3**: *Wdr47* loss leads to cell death independently of autophagy induction, related to Figure 5. .... Page 8
- **Appendix Table S1**: List of primary and secondary antibodies used in this work. .... Page 10
- **Appendix Table S2**: List of RT-qPCR primers used in this work. .... Page 11

Appendix Note 1: **Clinical features of patients with *WDR47* variants, related to Figure 1.**

Patients M01/M02 (c.578G>A, p.Arg193His)

Two affected newborns showed severe microcephaly with overlapping sutures, brain underdevelopment evident by remarkable simplified gyral pattern, agenesis of corpus callosum and pontocerebellar hypoplasia.

The patient M01 was born to a healthy consanguineous Sudanese couple. The pregnancy was normal. Abnormal rapid brief and violent intrauterine movement (intrauterine convulsions) were noticed at 20 weeks' gestation. Abnormal brain was noted at 20 weeks. Reduced Occipito-Frontal Circumference (OFC) with dilated ventricular system was noticed at 20 weeks' gestation in anti-natal scan. Polyhydramnios was noted at 22 weeks. At 26 weeks' gestation, microcephaly, dilated ventricular system with thin cerebral mantle and abnormal gyral pattern, hypoplastic cerebellum and corpus callosum agenesis were confirmed. Amniocentesis was done at 30 weeks due to massive polyhydramnios. Delivery was via elective Caesarean section (C-section) at 37 weeks. Weight at birth was 2.5kg, length average with OFC of 32cm. No obvious facial dysmorphism was observed. Other systems examination and skin were unremarkable. Myoclonic convulsions were seen immediately after birth. Death was at 24 hours.

The M02 patient was born after a normal pregnancy. Abnormal rapid brief and violent intrauterine movement (intrauterine convulsions) were noticed at 20 weeks' gestation. Abnormal brain was noted at 20 weeks. Anti-natal ultrasound at 20th week showed reduced OFC and dilated ventricular system. Polyhydramnios was noted at 22 weeks. At 26 weeks' gestation, anti-natal ultrasound revealed microcephaly, dilated ventricular system, thin cerebral mantle with abnormal gyral pattern, hypoplastic cerebellum and corpus callosum agenesis. Delivery was via elective C-section at 36 weeks due to massive polyhydramnios. Weight at birth was 2.3kg, length average with OFC 32cm. Myoclonic convulsions was seen immediately after birth. The baby received a loading dose of phenobarbital with no response. No mechanical ventilation was needed. Oxygen saturation was maintained by Oxygen mask. No abnormal breathing was documented. Death was at 48 hours. No autopsy was done on either baby.

Patient M03 (c.1396G>C, c.1775A>G; p.Asp466His, p.Lys592Arg)

Eleven-year-old boy showed growth failure, postnatal microcephaly, periventricular heterotopia, dysmorphisms, and severe neurodevelopmental delay.

The patient M03 was the third child born to healthy, unrelated Japanese parents. The pregnancy was uneventful, and he was delivered spontaneously with no asphyxia at 38 weeks of gestation. His birth weight was 2772g, occipito-frontal circumference (OFC) was 31.5cm (-1.1 SD). The neonatal period was marked by frequent vomiting, poor feeding and failure to thrive followed by severe growth failure.

Neurodevelopmental delays were noted, such as head control at 3 years of age, hand-supported sitting at 8

years. At 11 years, his height was 123.0cm (-3.4 SD), weight was 11.79kg (-3.1 SD) and OFC was 45.0 cm (-5.9 SD). He showed widely spaced eyes, epicanthus, tented upper lip, and short philtrum, pes cavus, and elevated right testis. He obtained no meaningful words and no ambulation. Brain Magnetic Resonance Imaging (MRI), at 9y9m, revealed bilateral periventricular heterotopia, mild dilatation of the lateral ventricles and thin corpus callosum. At 11y11m, M03 had encephalopathy. He had fever, seizures, and impaired consciousness. Electroencephalography showed slow waves in the left hemisphere. Magnetic Resonance Spectroscopy (MRS) showed high glutamate levels. He was treated with mPSL (methylprednisolone) for 3 days, mannitol and Phenytoin (PHT), discharged on day 15 with no abnormalities on MRI. He is currently continuing on Levetiracetam medications. At 12 years, gastrointestinal endoscopy was performed due to occasional brownish vomiting and moodiness, which revealed severe esophagitis. After undergoing surgery, he is doing well and gaining weight.

Patient M04 (c.1949C>T; p.Pro650Leu)

Patient M04 was delivered via lower segment cesarian. He was admitted to neonatal intensive care unit for 42 days. He had an abnormal neurological evaluation and displayed abnormal development with seizure of resistant type, decreased activity and spontaneous movement, poor sucking, and reduced weight gain. His muscle tone was hypotonic with poor head control and exaggerated tendon reflexes, which progressed with his neurological disease. At 12-month-old, he presented with microcephaly, persistent head lag, abnormality of ocular smooth pursuit, myoclonic seizure and hyperreflexia. His follow-up assessment at 3y3m revealed severe neurodevelopmental retardation and semi-vegetative condition, with resistance seizures requiring three anti-convulsant medications. He was bedbound with very limited spontaneous movement. He was not able to stand or respond to any command and did not speak any words. His joints was stiff with generalized muscle mass reduction and significant reduction in physical growth with microcephaly. His weight was 10.5kg (-2.98 SD), length was 92cm (-1.32 SD), and head circumference was 44cm (-3.7 SD).

The brain MRI performed at 1y showed the brain parenchymal volume loss and rather thin hypoplastic corpus collosum and extensive periventricular cystic changes consistent with leukomalacia.

The clinicopathological assessment for this child rather revealed an essential developmental process perturbation affecting primarily the brain and central neurological system. There was no metabolic decompensation observed, and the abdominal ultrasound reported unremarkable for abdominal organs (normal spleen, liver and pancreas) except some dilatation of renal pelvises but no cystic lesions. There was no evidence of situs inversus or heterotaxy on the chest X-ray and cardiac echocardiography.

Patient M04 older sibling died at 6 years old with severe neurodevelopmental retardation, microcephaly, and seizures.

Patients M05/M06 (c.1949C>T; p.Pro650Leu)

Both siblings are affected by severe psycho-motor retardation. Parents are clinically healthy and apparently unrelated; a distant consanguinity cannot be excluded as they are members of a small community (1,700 inhabitants) in the geographical area of Cilento (Italy) with a high inbreeding coefficient. Familiar anamnesis was positive for the presence a similar syndrome in a paternal first cousin, who died at the age of 3 years; distant consanguinity was also probable for the parents of the cousin. We do not have any genetic documentation for a precise comparison between the cousin and our probands.

M05 was born at 40 weeks of gestation by C-section, due to lack of cervical dilatation. The pregnancy history was negative for exposure to potentially teratogenic factors in the first trimester. Birth parameters were 3529g of weight (75th centile), length of 52cm (75-90th centile), and head circumference of 35cm (50th centile). M05 presented with sucking difficulties and a weak cry, and head control was achieved only at the age of 5 months. A transfontanellar ultrasound performed at birth and at 50 postnatal days showed hypoplasia of the corpus callosum, which was confirmed by a brain MRI. The MRI also revealed hypotrophy of the pons and midbrain, colpocephaly, supratentorial cortical atrophy, and diffuse hypomyelination with a quantitative reduction of white matter. In the hypothesis of Rett Syndrome, genomic tests were carried out to check for deletions or duplications in the genes most frequently involved in its etiology (*MECP2*, *CDKL5*, *FOXG1*, *MEF2C)*. The cytogenetic investigation on the peripheral blood of M05 showed a normal karyotype, as did both parents. Array-CGH analysis using a 180K platform did not reveal any genomic imbalances with known pathogenic effects. At the 1 year and 5 months follow-up, M05's head circumference was 42.9cm (3-10th centile), length was 78cm (90th centile), and weight was 8.6kg (25-50th centile). A peculiar facial appearance was also evident, including a high labial philtrum, anteverted nares, a depressed nasal root, and maxillary hypoplasia. Currently, the girl is 9 years old, and the psychomotor retardation remains very severe, with no improvement and no control of the sphincters.

Two years later, a second child was born, and all milestones of psychomotor development were normal. Currently, at 7 years of age, the child is perfectly normal.

M06 was born, at 40 weeks of gestation by C-section. Birth parameters were 3350g of weight (50th centile), length of 51cm (75-90th centile), and head circumference of 35cm (50th centile). The clinical phenotype and psychomotor development were identical to those of the eldest sister M05. The identification of the syndrome accelerated the diagnostic process, which confirmed severe psychomotor retardation with epileptic manifestations, hypoplasia of the corpus callosum, cerebral atrophy, and secondary microcephaly (not present at birth).

Patient M07 (c.1976A>C; p.His659Pro)

The proband is now a 4-year-old male from the Mina Gerais state, the first and only child born to healthy, consanguineous parents (first cousins), with no additional relevant family history. Pregnancy was complicated by gestational diabetes mellitus starting in the 6th month and was managed with dietary treatment, resulting in adequate glycemic control. Fetal movements were reported as normal, but a second

trimester morphology ultrasound disclosed fetal ventriculomegaly. Subsequent obstetric ultrasounds toward the end of the third trimester showed that the fetal head circumference was on the lower limit. He was born at term by C-section, with an Apgar score of 9/9, a weight of 3020g, a length of 50.8cm, and a head circumference of 33cm. On the first day of life, he was admitted to the Neonatal Intensive Care Unit (NICU) due to poor sucking and a hypoglycemic episode. An orogastric tube was inserted because of feeding difficulties. He remained in the NICU for observation, during which a transfontanellar ultrasound was performed, revealing corpus callosum dysgenesis. He was released from the maternity ward on the 7th day of life.

At three months of age, the parents noted spasms, and he was diagnosed with West syndrome after neurological evaluation and electroencephalography. Anticonvulsant therapy with Levetiracetam was initiated, but it did not produce an adequate response. An adrenocorticotropic hormone (ACTH) treatment course was performed, resulting in some improvement in the frequency of epileptic episodes; nonetheless he continues to experience almost daily epileptic crisis and is currently receiving Vigabatrin and Canabidiol. He has also undergone a second course of ACTH.

Additionally, around the same time, frequent vomiting and significant feeding difficulties with very poor weight gain were noticed. He was diagnosed with cow's milk protein allergy and gastroesophageal reflux disease, and underwent Nissen fundoplication and gastrostomy tube placement at 5 months of age, leading to subsequent adequate weight gain.

His neuropsychological development was severely delayed from the 4th month of life. Currently, he can only hold his head up for a brief period, cannot sit with or without support, does not speak and only makes sounds. At his last physical and neurological evaluation, at 2 years and 5 months, he presented a length in the 80th centile (+0.85 SD), a weight in the 70th centile (+0.54 SD), and a head circumference in the 4th centile (-1.73 SD). He also exhibited axial hypotonia, appendicular hypertonia, and signs of dystonia.

Brain MRI at 1 year of age disclosed a head circumference in the lower limit, reduced white matter, a hypoplastic corpus callosum, reduced brainstem volume, ventriculomegaly without hydrocephalus, and nonspecific hypersignal areas in the white matter.

Additional investigations included an ophthalmological exam showing significant strabismus, which was treated with botulinum toxin injections, and a normal ocular fundoscopy; normal hearing screening tests (Otoacoustic emissions - OAE); an abdominal and urinary tract ultrasound with no abnormalities; and a normal cardiological evaluation.

Appendix Figure S1: Patients with *WDR47* variants, related to Figure 2.

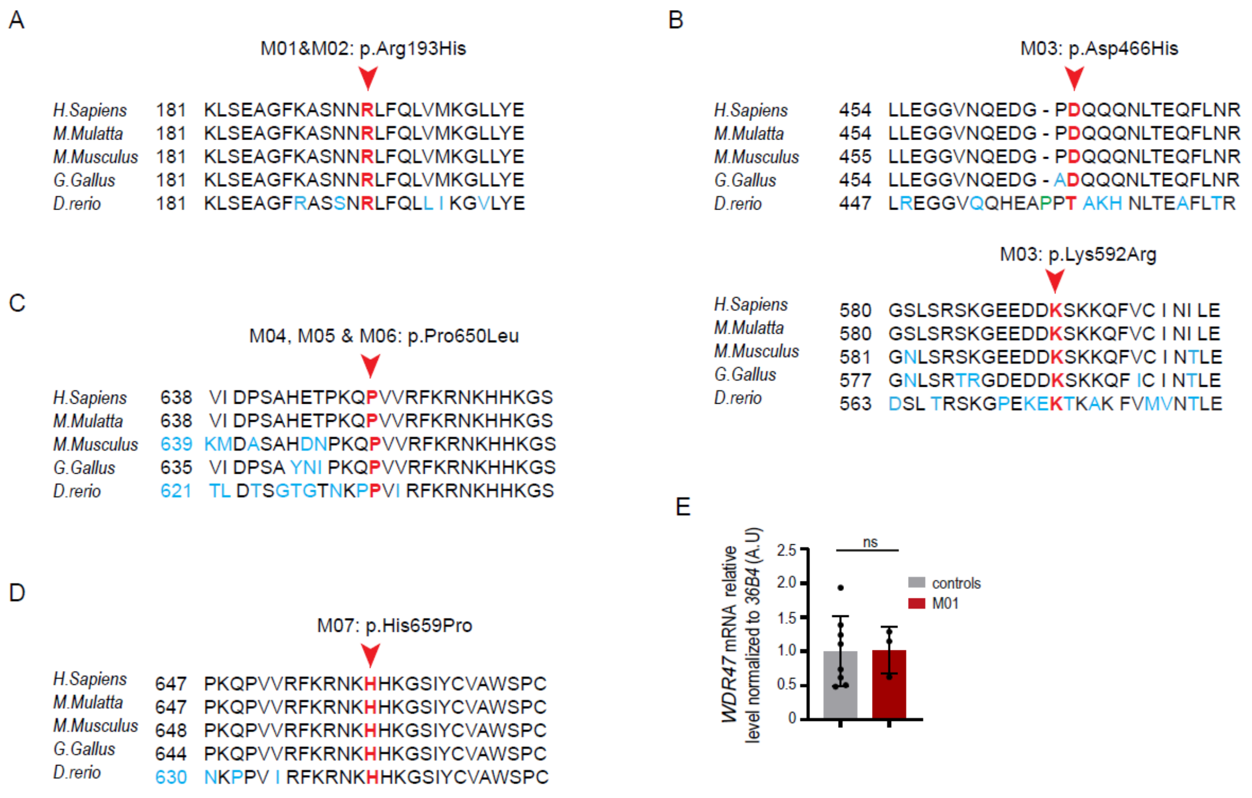

(A-D) Alignment of WDR47 protein across several species (human, macaque, mouse, fish, chicken and zebrafish) shows the conservation of mutated amino acid residue (red arrow head) in patients. **(E)** RT-qPCR analyses showing unchanged *WDR47* mRNA levels in M01. Each dot represents one independent measure and data from two individuals used as controls are pooled. Data (means ± s.d) were analyzed by unpaired two-tailed Student t-test, ns, non-significant.

Appendix Figure S2: Validation of the mouse models used in the study, related to Figure 3.

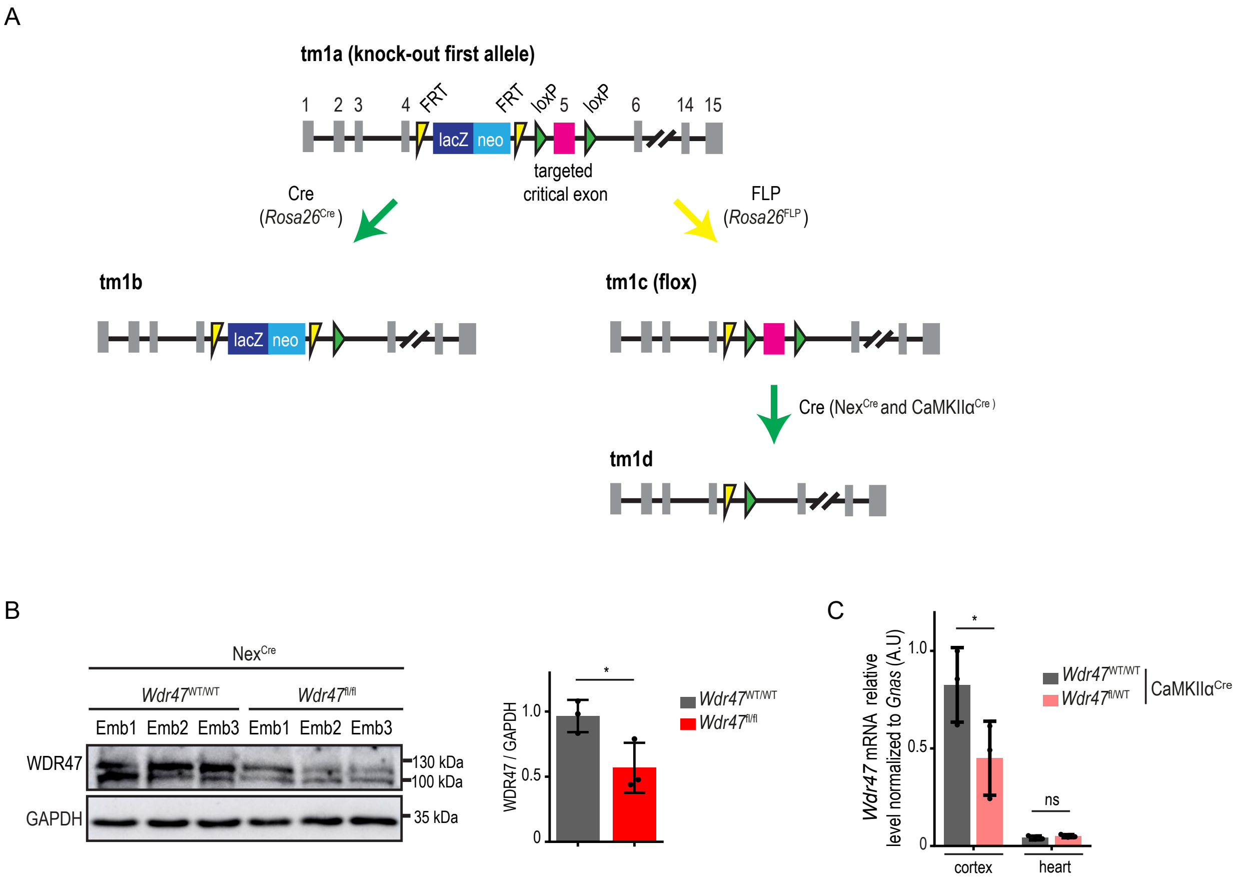

(A) Schematic showing allelic construction of the different *Wdr47* mouse models used in the study. FRT, flipase site; LacZ, β-galactosidase gene; loxP, Cre recognition site; neo, neomycin-resistant gene.

(B) Western blot analysis of WDR47 in cortical extracts from E18.5 embryos upon deletion of *Wdr47* in early glutamatergic neurons using the Nex^{Cre} mouse line. Gapdh is used as the loading control. Data (means ± sd) from 3 embryos per condition were analyzed by unpaired two-tailed Student t-test. **(C)** RT-qPCR analyses of *Wdr47* mRNA levels in cortical and heart extracts from adult mice upon deletion of *Wdr47* in post-mitotic neurons using the CaMKIIα^{Cre} line. Data (means ± s.d) from three samples per condition were analyzed by two-way ANOVA, with Bonferroni's multiple comparisons test. ns, non-significant; *P < 0.05.

Appendix Figure S3: *Wdr47* loss leads to cell death independently of autophagy induction, related to Figure 6.

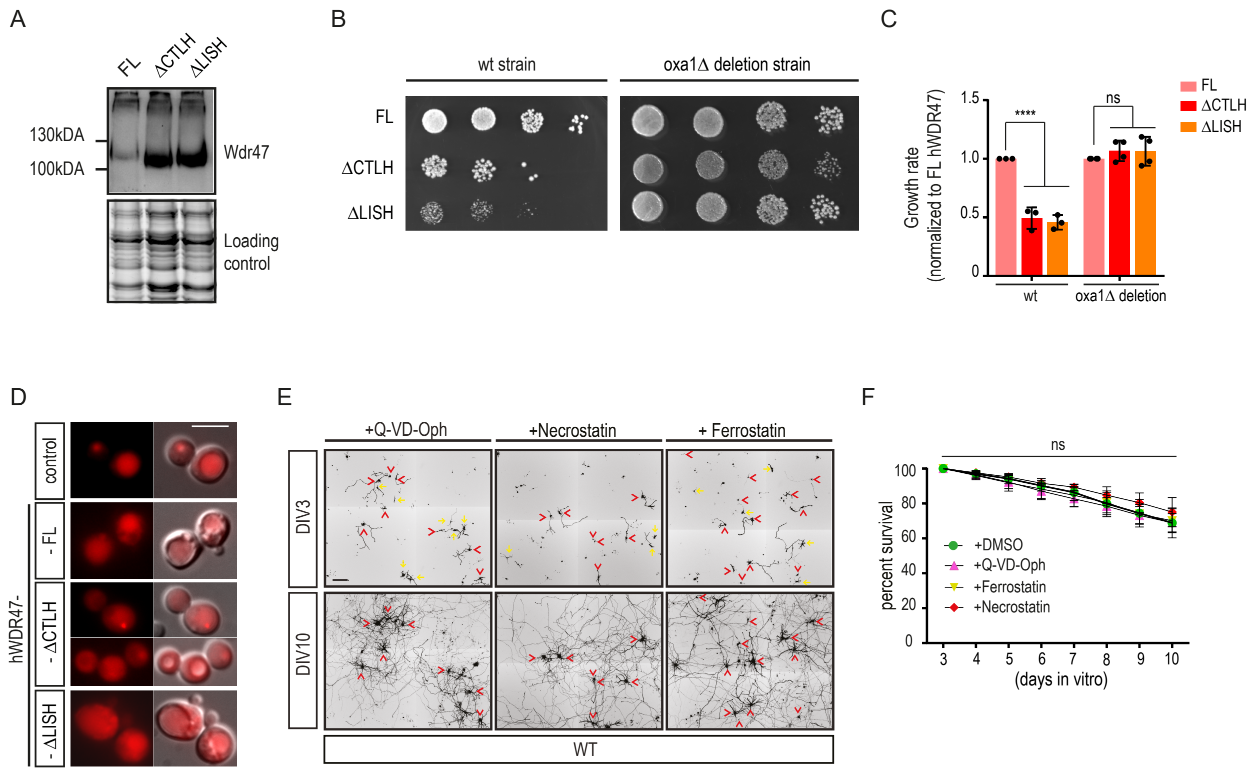

(A) WB analysis using yeast extracts showing expression of different hWDR47 constructs (full-length, ΔCTLH and ΔLISH). 2,2,2-Trichloroethanol (TCE) staining is used as loading control. **(B-C)** Yeast drop test growth assay. **(B)** The growth of wt (left panel) and oxa1 mutant (right panel) yeast strains expressing different Wdr47 constructs. **(C)** Quantification of yeast growth expressing different hWDR47 constructs for wt versus oxa1 deletion strains. Note that yeast growth is inhibited upon expression of ΔCTLH and ΔLISH constructs in wt but not in oxa1 deletion strains. Data (means ± sd) from at least 3 independent experiment per condition was analyzed by one-way ANOVA, with Bonferroni's multiple comparison test. **(D)** Fluorescent images of yeast cells, transformed with mCherry-Atg8 plasmid together with or without different WDR47 constructs. Yeast cells were incubated for 4 h in nitrogen starvation medium (SD-N) to induce autophagy. Note that mCherry signal which is strictly localized to the vacuole in control condition becomes diffuse and localizes both to vacuole and cytoplasm upon expression of different WDR47 constructs. Scale bar: 5 μm. **(E-F)** Effect of drugs used for rescue experiments on WT neuronal cultures. **(E)** Representative fields, at DIV3 and DIV10, of WT neuronal cultures treated with Qvd-OPh (50μM) (Caspase inhibitor), Ferrostatin (5 μM) (ferroptosis inhibitor) and Necrostatin (2 μM) (Necroptosis inhibitor) at DIV2. Scarlet positive electroporated neurons are depicted in

black. Yellow arrows correspond to neurons that died, red arrowheads correspond to neurons that are alive and could be followed from DIV3 to DIV10. **(F)** Survival of WT neurons from DIV3 to DIV10 upon different drug treatments. Data (means ± sd) from at least 3 independent cultures per condition was analyzed by two-way ANOVA, with Bonferroni's multiple comparison test. ns, non-significant, ****$P < 0.0001$

Appendix Table S1: List of primary and secondary antibodies used in this work.

1st antibody	Host	Dilution	Used for	Company	Reference
Anti-activated Caspase-3	Rabbit	1/100	IHC	R and D system	AF835
Anti-GFP	Chicken	1/800	IHC	Aves lab	GFP-1020
Anti-LAMP-1	Mouse	1/1000	ICC	Developmental Studies – Hybridoma Bank	H4A3
Anti-WDR47	Rabbit	1/200 (human)<br>1/2000 (mouse)	IHC	Atlas antibodies	HPA027289
Anti-GAPDH	Mouse	1/5000	WB	Sigma-Aldrich	MAB-374
Anti-GFP	Goat	1/1000	WB		
Anti-α-tubulin	Mouse	1/2000	WB	Sigma-Aldrich	T9026
Anti-acetylated α-tubulin	Mouse	1/500	WB	Thermo Fisher	322700
Anti-tyrosinated α-tubulin	Rat	1/2000	WB	Sigma-Aldrich	MAB1864-I
Anti-β actin coupled HRP	Mouse	1/40.000	WB	Sigma-Aldrich	A3854
Anti-HA	Rat	1:2000	WB	Roche Life Science products	11867423001
Anti-WDR47	Rabbit	1/1000	WB	Abcam	ab121935
**2nd antibody**	**Host**	**Dilution**	**Used for**	**Company**	**Reference**
Anti-chicken-488	donkey	1/1000	IHC	Abcam	ab63507
Anti-rabbit-647	donkey	1/1000	IHC	ThermoFisher Sc.	A-31573
Anti-mouse-555	donkey	1/1000	ICC	ThermoFisher Sc.	A-31570
mouse-HRP	Goat	1/10 000	WB	ThermoFisher Sc.	G-21040
rabbit-HRP	Goat	1/10 000	WB	ThermoFisher Sc.	G-21234
rat-HRP	Goat	1/10 000	WB	ThermoFisher Sc.	62-9520
goat-HRP	Donkey	1/5000	WB	Santa Cruz	Sc2020

ICC,immunocytochemistry; IHC, immunohistochemistry; WB, western blot.

Appendix Table S2: List of RT-qPCR primers used in this work.

Gene	Species	Forward sequence	Reverse sequence
Actin	mouse	TATAAAACCCGGCGGC	TCATCCATGGCGAACTGGTG
Dync1l1	mouse	AGGAAGTGATCGCTCCGGG	CAGACATGTTTGCTTCCTGAATG
Klc2	mouse	CCGTTCCCAGTGGTGGTATC	CTCCAAGACCAGGCGAAGAG
Ndufs7	mouse	GTGTCCATGGGGAGCTGTG	CCATAAAGGAGTGCTTCGGC
Nefl	mouse	CCGGGGTATGAACGAAGCTC	CAGTTTGTTGATTGTGTCCTGC
Sod1	mouse	GGGAAGCATGGCGATGAAAG	GGTTCACCGCTTGCCTTCTG
Trap1	mouse	GTCCCGGGTACAAGATGTGG	CATCCAGATGGCCTGCAAAG
Tuba4A	mouse	CTACGTGAGACGTACAGCCC	TGGTCTTATCGCTGGGCATC
Tubb4A	mouse	AGAGGAGGCTGAAGAGGAGG	TGGGGTCCTAGGGAATGAGG
Tubb4B	mouse	TCTACAGCTGTTCCGCAGTC	CTCGTCGCTGATTACCTCCC
Uqcrq	mouse	TAGGCCGTGGGAGGTTTTTC	AAAGGGCGACAAGCTGTAGG
Atf4	mouse	GGTGGCCAAGCACTTGAAAC	CGGAAAAGGCATCCTCCTTG
Wdr47	mouse	GGACCCCAGTGGCCGTCTCT	GCTCTTTCTGGGGCAGGACGC
GNAS	mouse	AGAACATCCGCCGTGTCTTC	CCTTCTTAGAGCAGCTCGTATTGG
WDR47	human	TCCTGAGGCAGCTAATACTTG	TCGCGTTGTTAACACATAAAGC
36B4	human	GCCTTGACCTTTTCAGTAAG	ATGGGTACAAGCGCGTCCTG
