# Supplementary material for: Bi-allelic variants in WDR47 cause a complex neurodevelopmental syndrome
Source: EMBO Mol Med. 2024 Nov 28;17(1):129–68. doi: 10.1038/s44321-024-00178-z (PMC11730659; doi:10.1038/s44321-024-00178-z)
Supplement: Supplementary file 9 — Source data Fig. 2 [file 44321_2024_178_MOESM9_ESM.zip › Figure2 new/2F/Western blot/Figure 2F with annotations .pptx]

## Slide 1
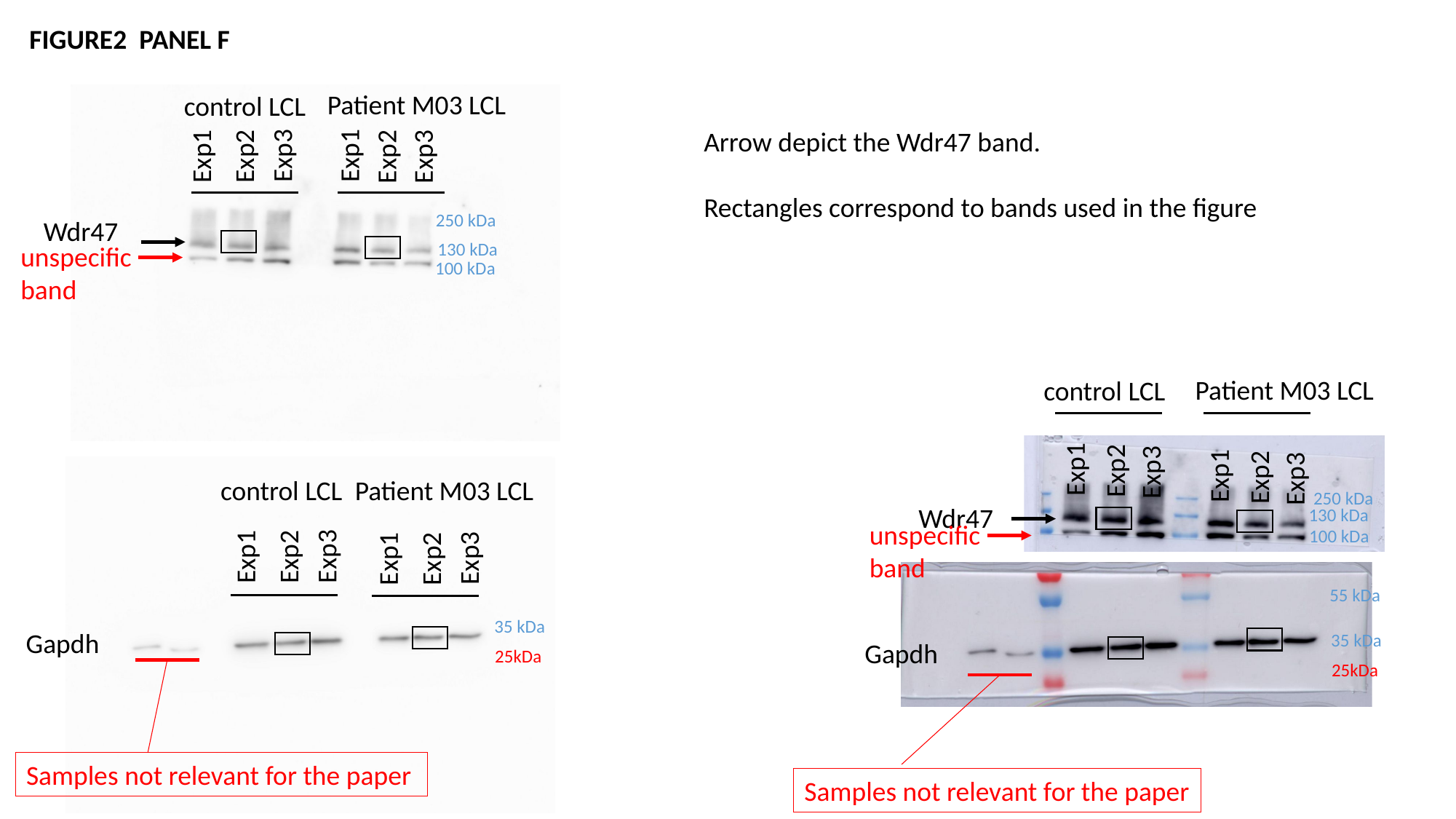

FIGURE2 PANEL F
Patient M03 LCL
control LCL
Arrow depict the Wdr47 band.
Rectangles correspond to bands used in the figure
Exp3
Exp1
Exp2
Exp1
Exp2
Exp3
250 kDa
Wdr47
130 kDa
unspecific
band
100 kDa
Patient M03 LCL
control LCL
Exp1
Exp2
Exp3
Exp1
Exp2
Exp3
Patient M03 LCL
control LCL
250 kDa
Wdr47
130 kDa
unspecific
band
100 kDa
Exp3
Exp2
Exp1
Exp3
Exp2
Exp1
55 kDa
35 kDa
Gapdh
35 kDa
Gapdh
25kDa
25kDa
Samples not relevant for the paper
Samples not relevant for the paper
