# Supplementary material for: Bi-allelic variants in WDR47 cause a complex neurodevelopmental syndrome
Source: EMBO Mol Med. 2024 Nov 28;17(1):129–68. doi: 10.1038/s44321-024-00178-z (PMC11730659; doi:10.1038/s44321-024-00178-z)
Supplement: Supplementary file 9 — Source data Fig. 2 [file 44321_2024_178_MOESM9_ESM.zip › Figure2 new/2E/Western blot/Figure 2E with annotations .pptx]

## Slide 1
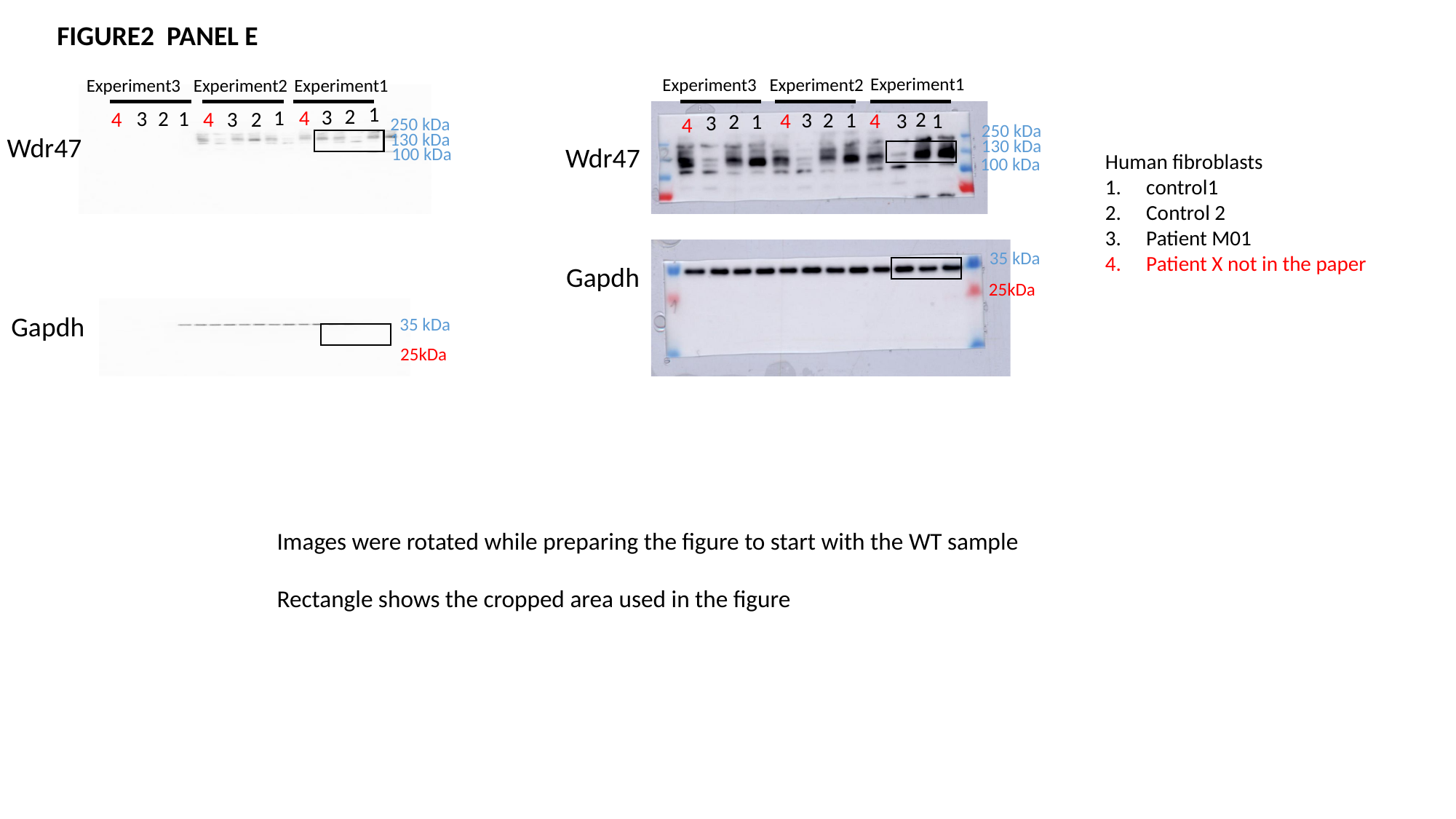

FIGURE2 PANEL E
Experiment1
Experiment3
Experiment2
Experiment1
Experiment3
Experiment2
1
2
3
1
4
3
2
1
4
3
2
4
2
3
2
1
4
3
1
4
2
1
3
4
250 kDa
250 kDa
130 kDa
Wdr47
130 kDa
Wdr47
100 kDa
Human fibroblasts
control1
Control 2
Patient M01
Patient X not in the paper
100 kDa
35 kDa
Gapdh
25kDa
Gapdh
35 kDa
25kDa
Images were rotated while preparing the figure to start with the WT sample
Rectangle shows the cropped area used in the figure
