# Supplementary material for: Bi-allelic variants in WDR47 cause a complex neurodevelopmental syndrome
Source: EMBO Mol Med. 2024 Nov 28;17(1):129–68. doi: 10.1038/s44321-024-00178-z (PMC11730659; doi:10.1038/s44321-024-00178-z)
Supplement: Supplementary file 9 — Source data Fig. 2 [file 44321_2024_178_MOESM9_ESM.zip › Figure2 new/2D/Western Blot/Figure 2D with annotations.pptx]

## Slide 1
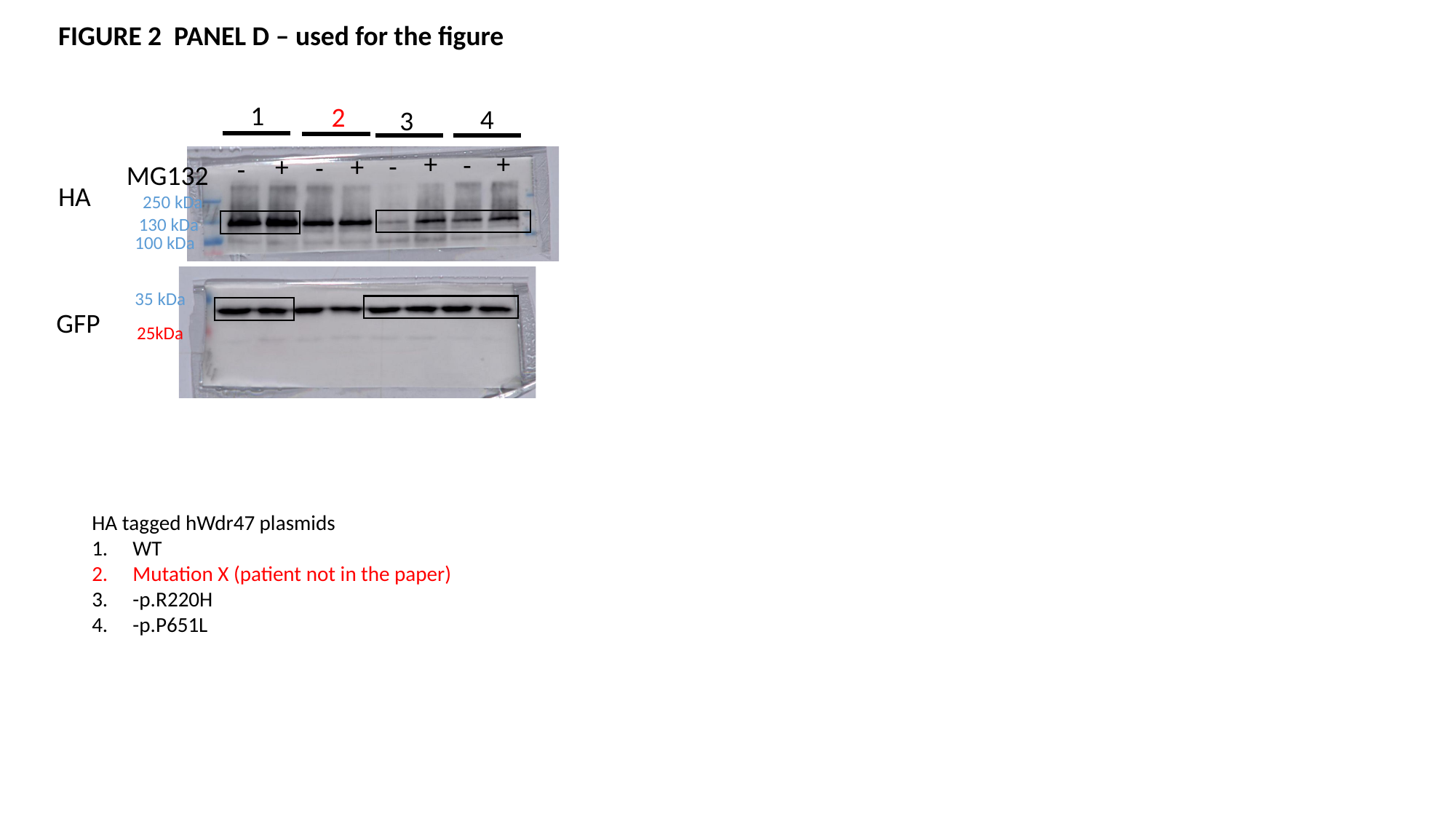

FIGURE 2 PANEL D – used for the figure
1
2
4
3
+
+
-
+
+
-
-
-
MG132
HA
250 kDa
130 kDa
100 kDa
35 kDa
GFP
25kDa
HA tagged hWdr47 plasmids
WT
Mutation X (patient not in the paper)
-p.R220H
-p.P651L

## Slide 2
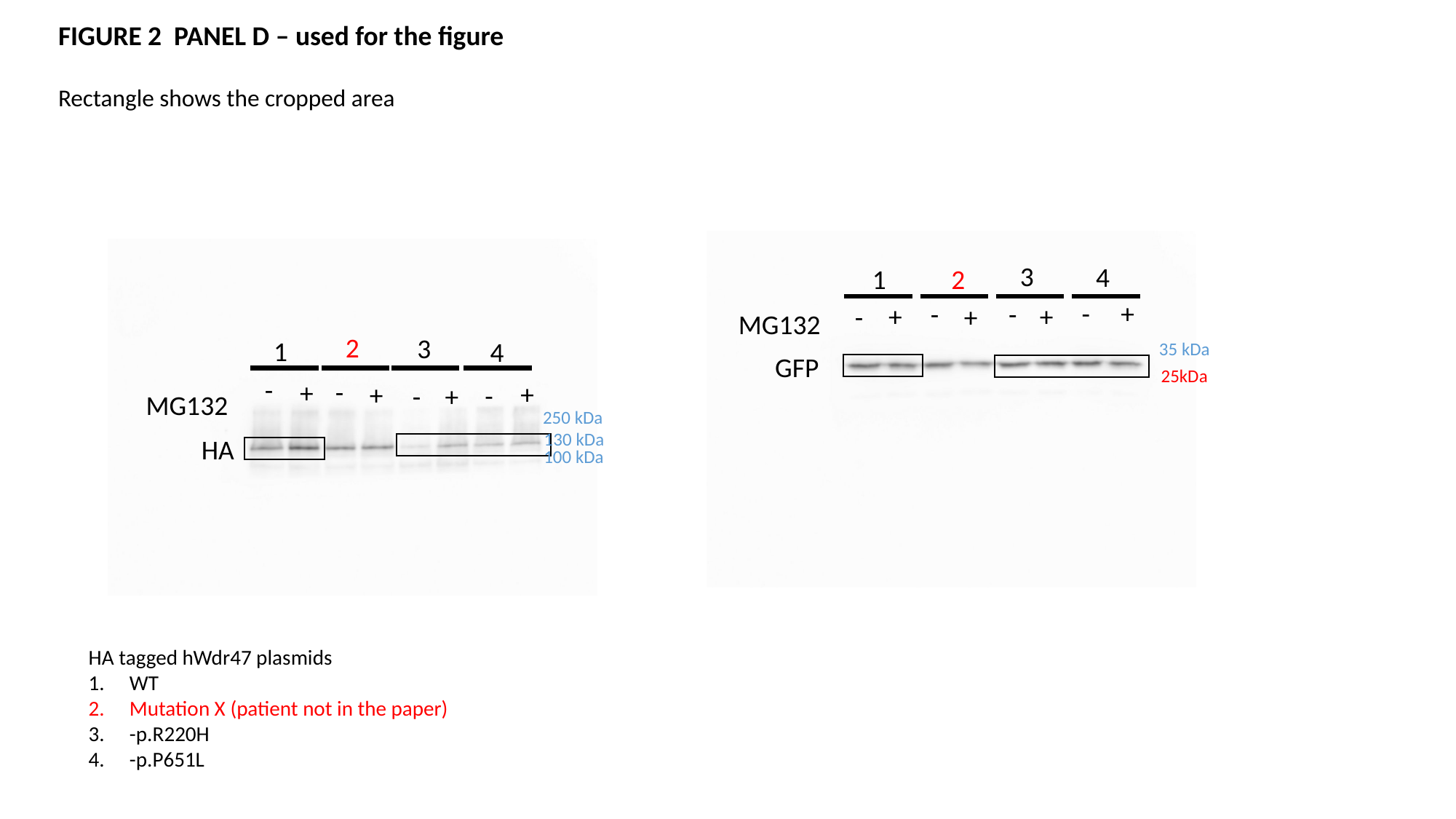

FIGURE 2 PANEL D – used for the figure
Rectangle shows the cropped area
3
4
1
2
-
+
-
-
+
+
-
+
MG132
2
3
1
4
35 kDa
GFP
25kDa
-
-
+
+
+
-
-
+
MG132
250 kDa
130 kDa
HA
100 kDa
HA tagged hWdr47 plasmids
WT
Mutation X (patient not in the paper)
-p.R220H
-p.P651L

## Slide 3
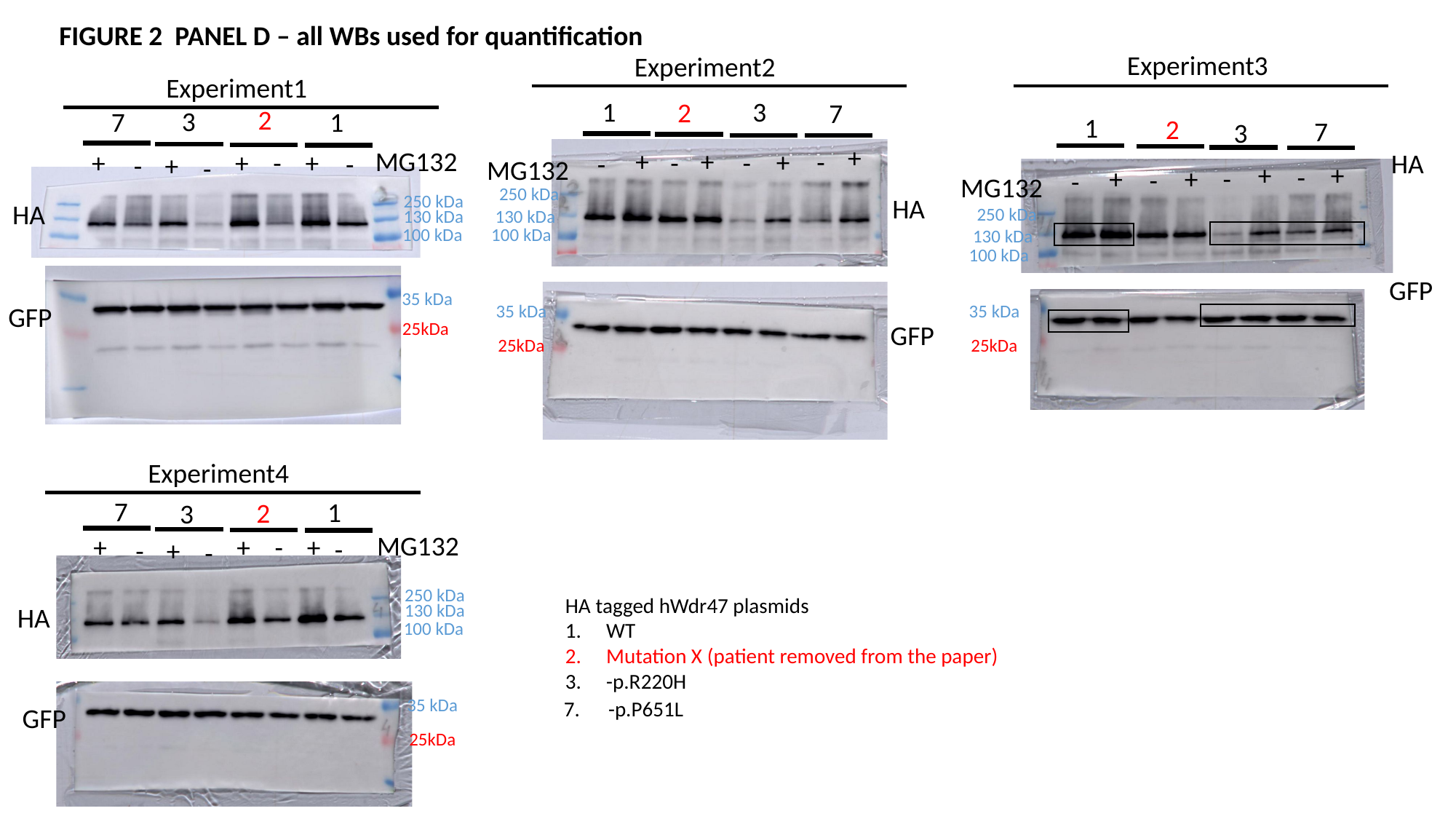

FIGURE 2 PANEL D – all WBs used for quantification
Experiment3
Experiment2
Experiment1
1
3
2
7
2
3
7
1
1
2
7
3
+
MG132
+
+
+
-
+
-
+
-
+
-
HA
-
-
-
+
-
MG132
+
+
-
+
+
-
-
-
MG132
250 kDa
250 kDa
HA
HA
250 kDa
130 kDa
130 kDa
100 kDa
100 kDa
130 kDa
100 kDa
GFP
35 kDa
35 kDa
35 kDa
GFP
25kDa
GFP
25kDa
25kDa
Experiment4
7
1
2
3
MG132
+
+
-
+
-
-
+
-
250 kDa
HA tagged hWdr47 plasmids
WT
Mutation X (patient removed from the paper)
-p.R220H
130 kDa
HA
100 kDa
35 kDa
7. -p.P651L
GFP
25kDa
