# Supplementary material for: Bi-allelic variants in WDR47 cause a complex neurodevelopmental syndrome
Source: EMBO Mol Med. 2024 Nov 28;17(1):129–68. doi: 10.1038/s44321-024-00178-z (PMC11730659; doi:10.1038/s44321-024-00178-z)
Supplement: Supplementary file 9 — Source data Fig. 2 [file 44321_2024_178_MOESM9_ESM.zip › Figure2 new/2C/Western blot/Figure 2C with annotations.pptx]

## Slide 1
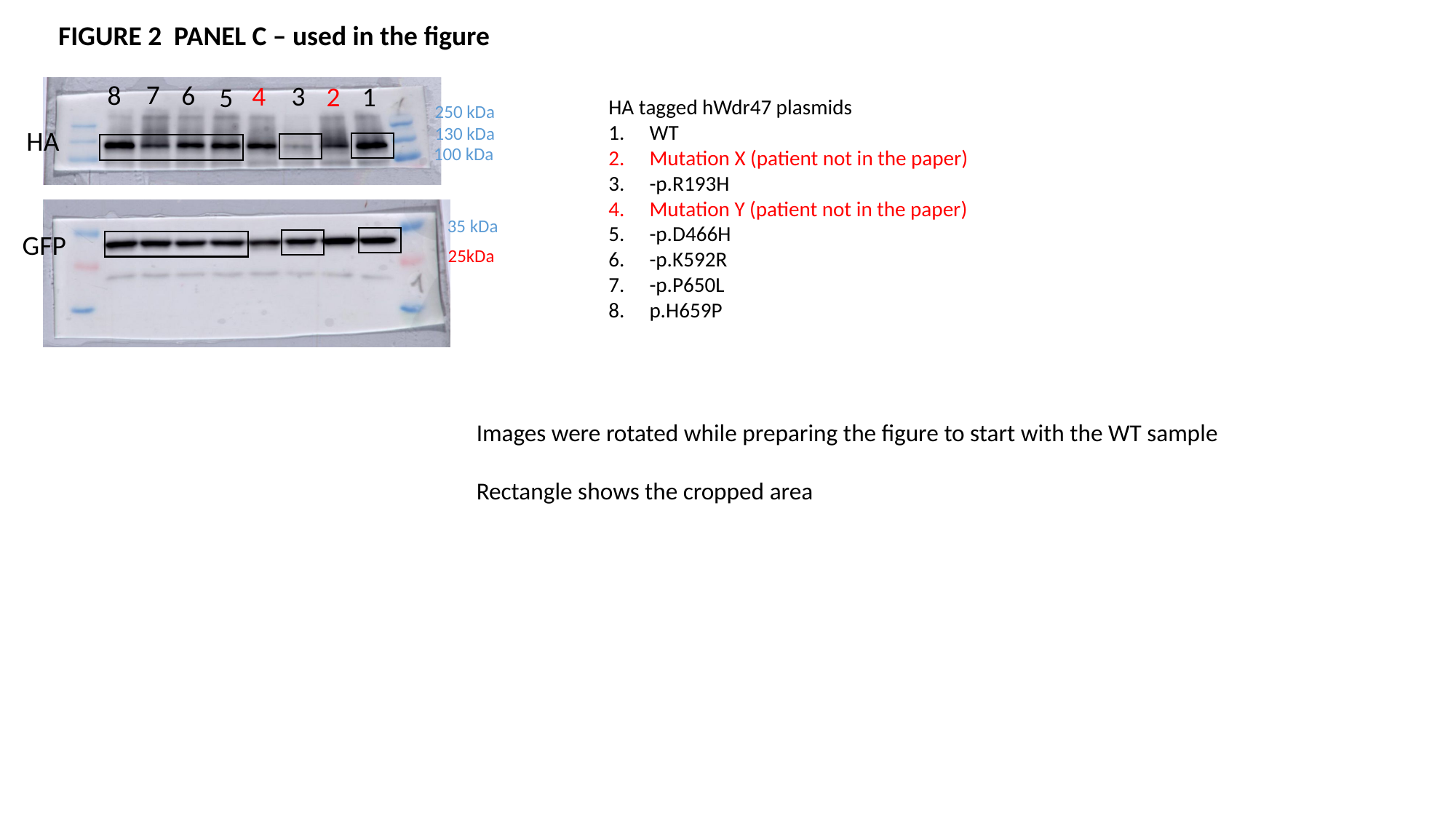

FIGURE 2 PANEL C – used in the figure
7
8
6
4
3
1
2
5
HA tagged hWdr47 plasmids
WT
Mutation X (patient not in the paper)
-p.R193H
Mutation Y (patient not in the paper)
-p.D466H
-p.K592R
-p.P650L
p.H659P
250 kDa
130 kDa
HA
100 kDa
35 kDa
GFP
25kDa
Images were rotated while preparing the figure to start with the WT sample
Rectangle shows the cropped area

## Slide 2
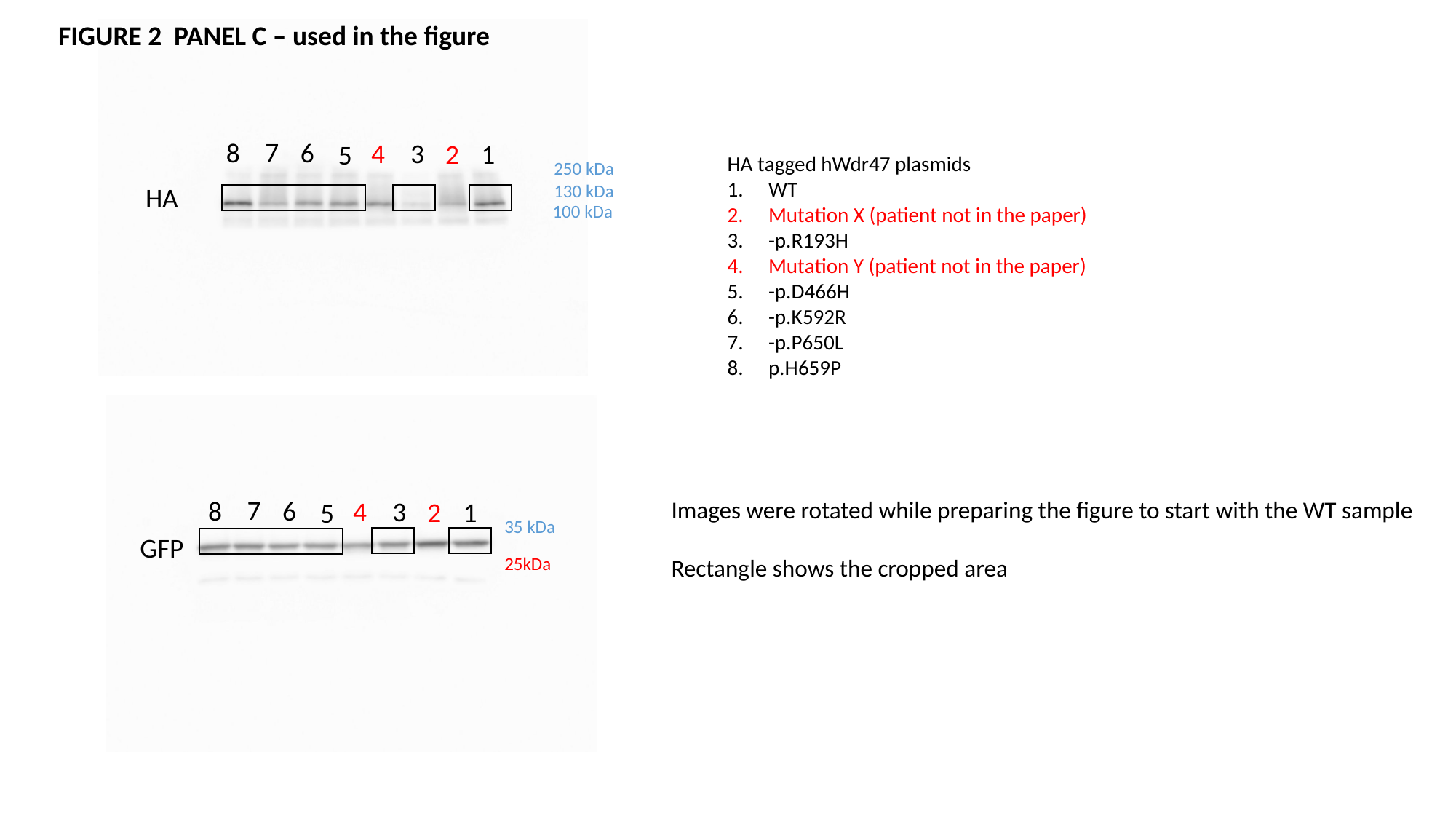

FIGURE 2 PANEL C – used in the figure
7
8
6
4
3
1
2
5
HA tagged hWdr47 plasmids
WT
Mutation X (patient not in the paper)
-p.R193H
Mutation Y (patient not in the paper)
-p.D466H
-p.K592R
-p.P650L
p.H659P
250 kDa
130 kDa
HA
100 kDa
7
8
6
4
3
Images were rotated while preparing the figure to start with the WT sample
Rectangle shows the cropped area
1
2
5
35 kDa
GFP
25kDa

## Slide 3
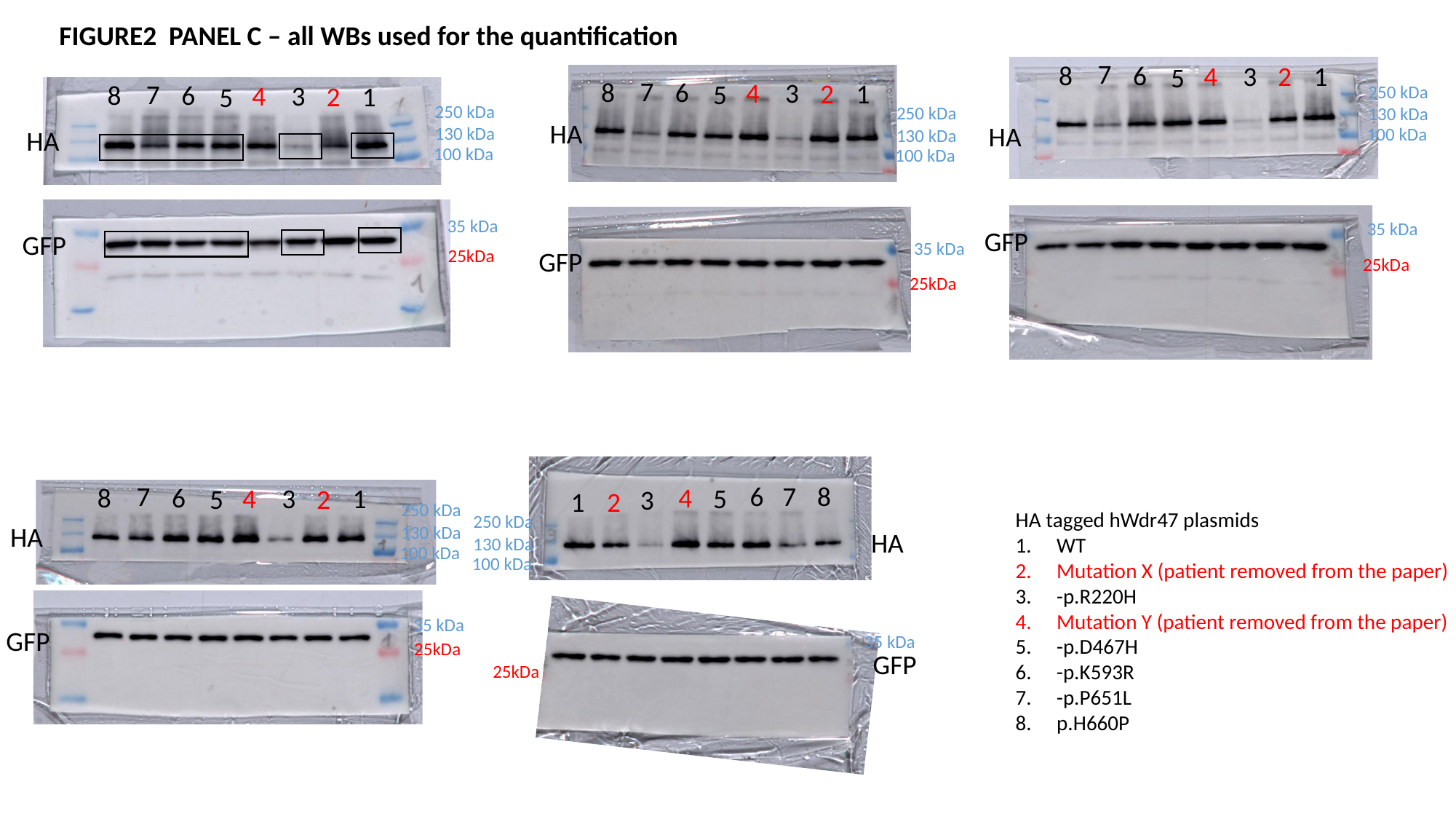

FIGURE2 PANEL C – all WBs used for the quantification
7
8
6
4
3
1
2
5
7
8
6
4
3
1
2
7
5
8
6
4
3
1
2
5
250 kDa
250 kDa
250 kDa
130 kDa
HA
HA
130 kDa
100 kDa
HA
130 kDa
100 kDa
100 kDa
35 kDa
35 kDa
GFP
GFP
35 kDa
25kDa
GFP
25kDa
25kDa
7
6
7
8
8
6
4
4
3
5
1
2
5
3
1
2
250 kDa
HA tagged hWdr47 plasmids
WT
Mutation X (patient removed from the paper)
-p.R220H
Mutation Y (patient removed from the paper)
-p.D467H
-p.K593R
-p.P651L
p.H660P
250 kDa
HA
130 kDa
HA
130 kDa
100 kDa
100 kDa
35 kDa
GFP
35 kDa
25kDa
GFP
25kDa
