# Supplementary figures and images for: Bi-allelic variants in WDR47 cause a complex neurodevelopmental syndrome
Source: EMBO Mol Med. 2024 Nov 28;17(1):129–68. doi: 10.1038/s44321-024-00178-z (PMC11730659; doi:10.1038/s44321-024-00178-z)

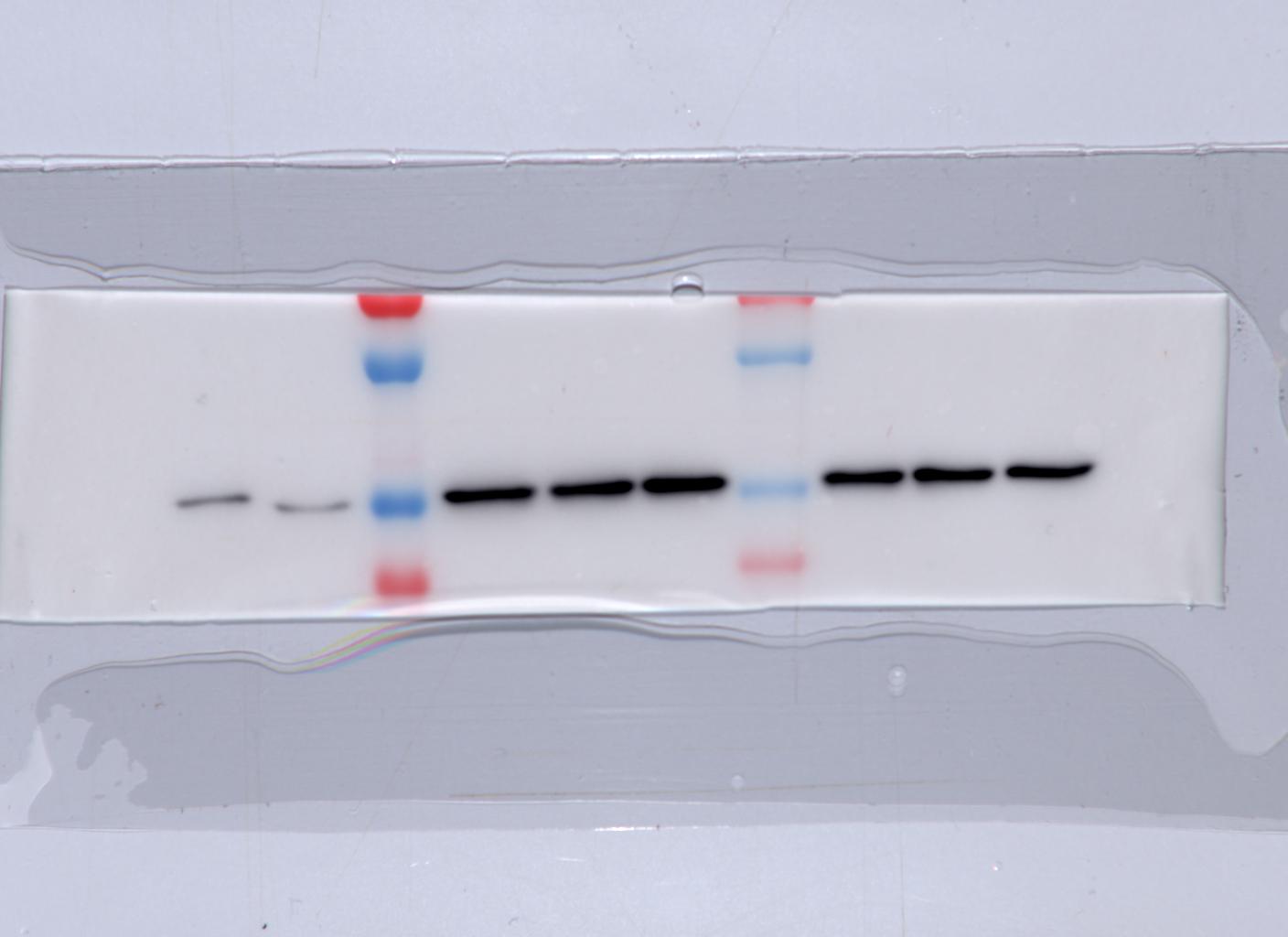

Supplement: Supplementary file 9 — Source data Fig. 2 [file 44321_2024_178_MOESM9_ESM.zip › Figure2 new/2F/Western blot/Gapdh/GAPDH #2 2023.09.27_14.19.02_Ch+Marker.jpg]

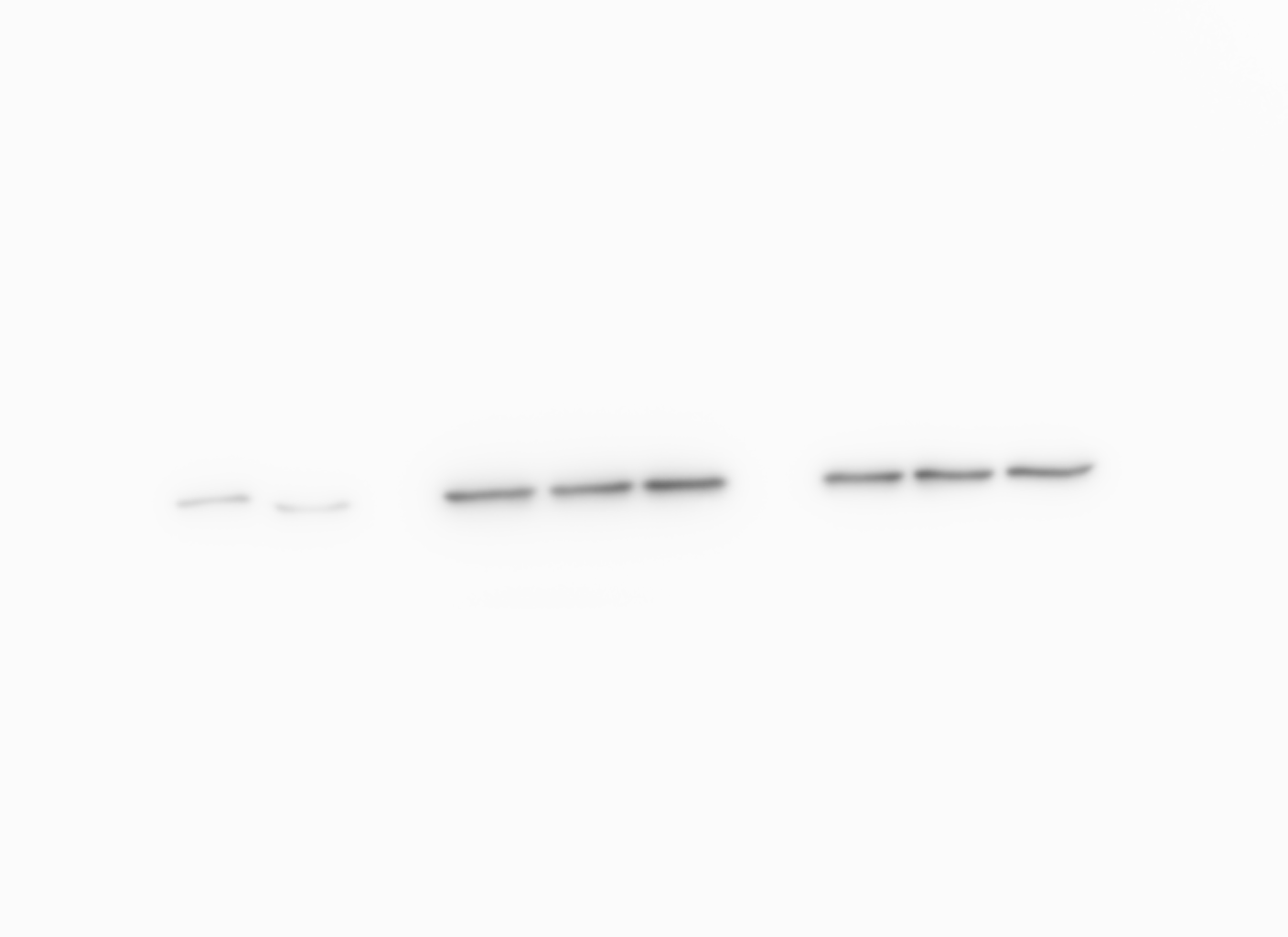

Supplement: Supplementary file 9 — Source data Fig. 2 [file 44321_2024_178_MOESM9_ESM.zip › Figure2 new/2F/Western blot/Gapdh/GAPDH #2 2023.09.27_14.19.02_Ch.tif]

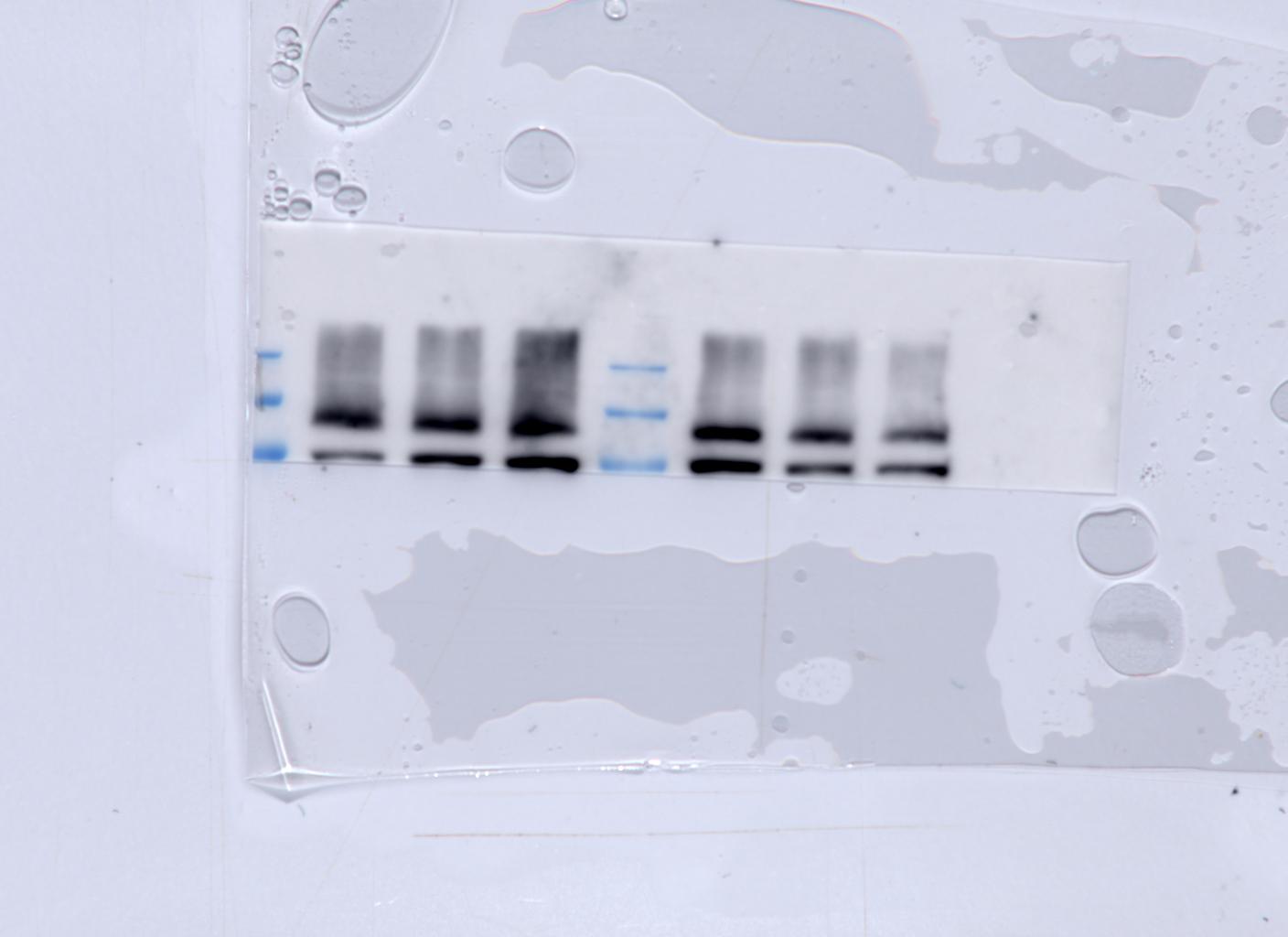

Supplement: Supplementary file 9 — Source data Fig. 2 [file 44321_2024_178_MOESM9_ESM.zip › Figure2 new/2F/Western blot/Wdr47/wdr47 CNT + MUT bis 2023.09.27_11.59.15_Ch+Marker.jpg]

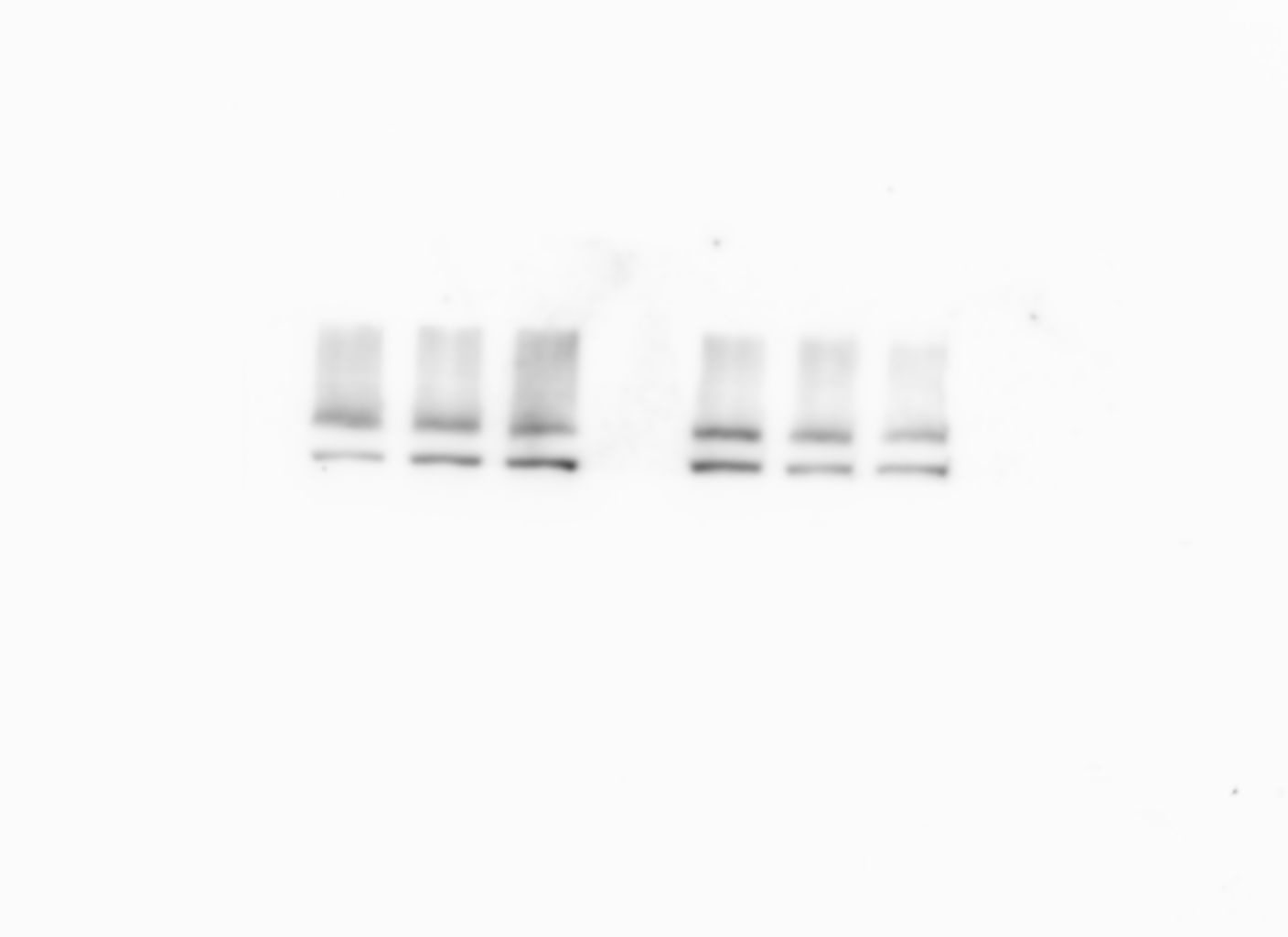

Supplement: Supplementary file 9 — Source data Fig. 2 [file 44321_2024_178_MOESM9_ESM.zip › Figure2 new/2F/Western blot/Wdr47/wdr47 CNT + MUT bis 2023.09.27_11.59.15_Ch.tif]

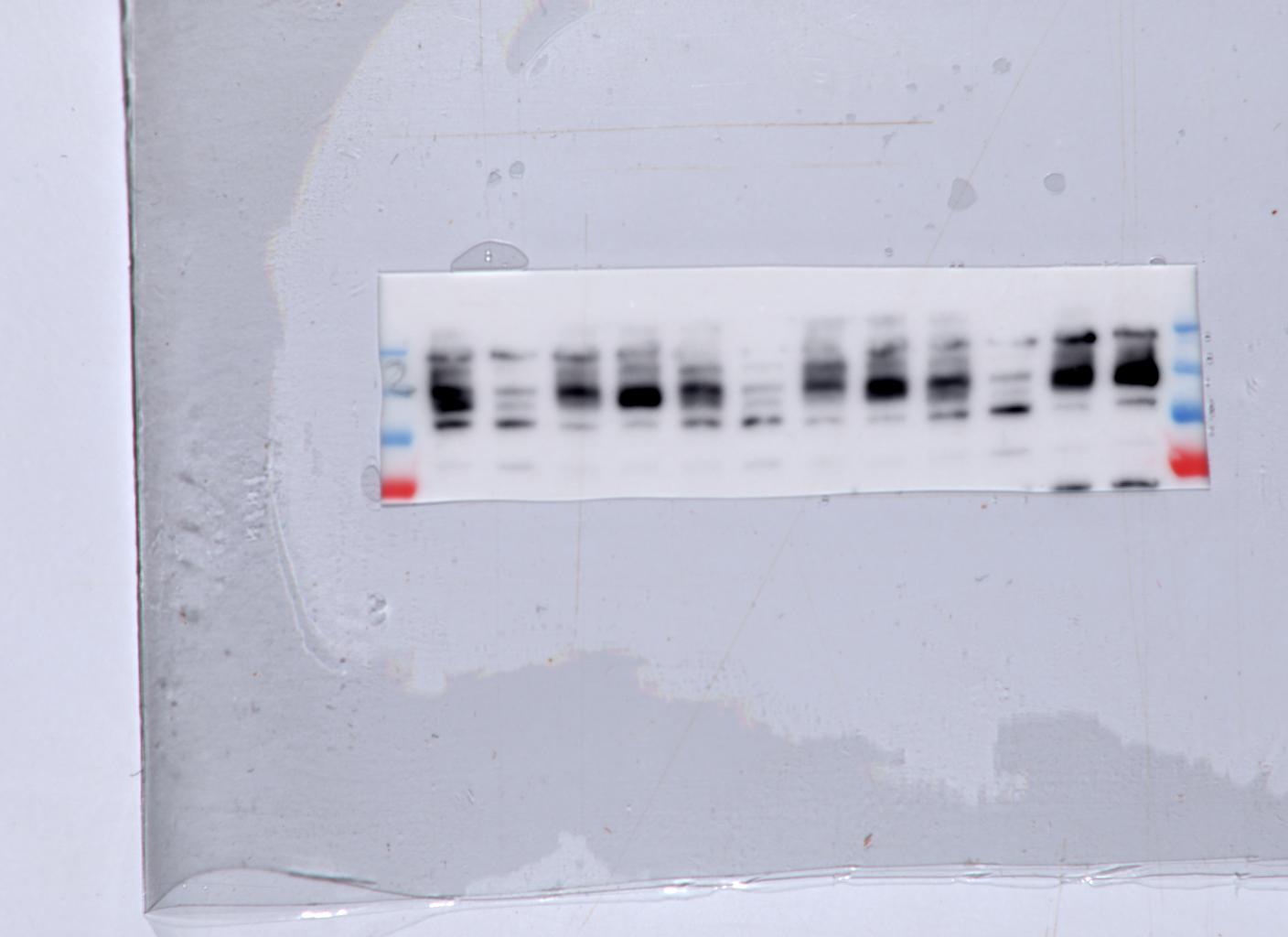

Supplement: Supplementary file 9 — Source data Fig. 2 [file 44321_2024_178_MOESM9_ESM.zip › Figure2 new/2E/Western blot/Wdr47/gel 2 wdr47 2023.04.13_11.41.43_Ch+Marker.jpg]

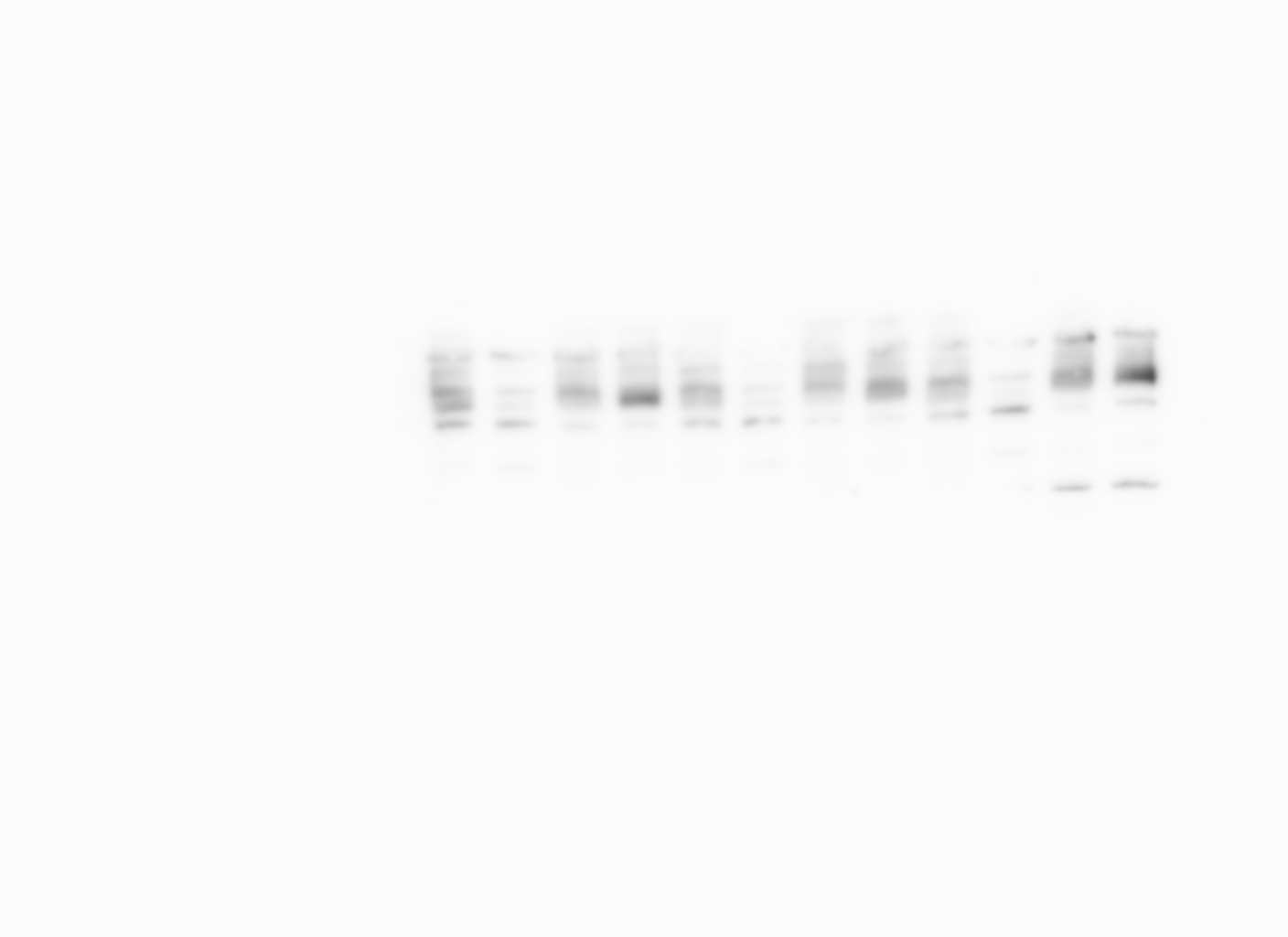

Supplement: Supplementary file 9 — Source data Fig. 2 [file 44321_2024_178_MOESM9_ESM.zip › Figure2 new/2E/Western blot/Wdr47/gel 2 wdr47 2023.04.13_11.41.43_Ch.tif]

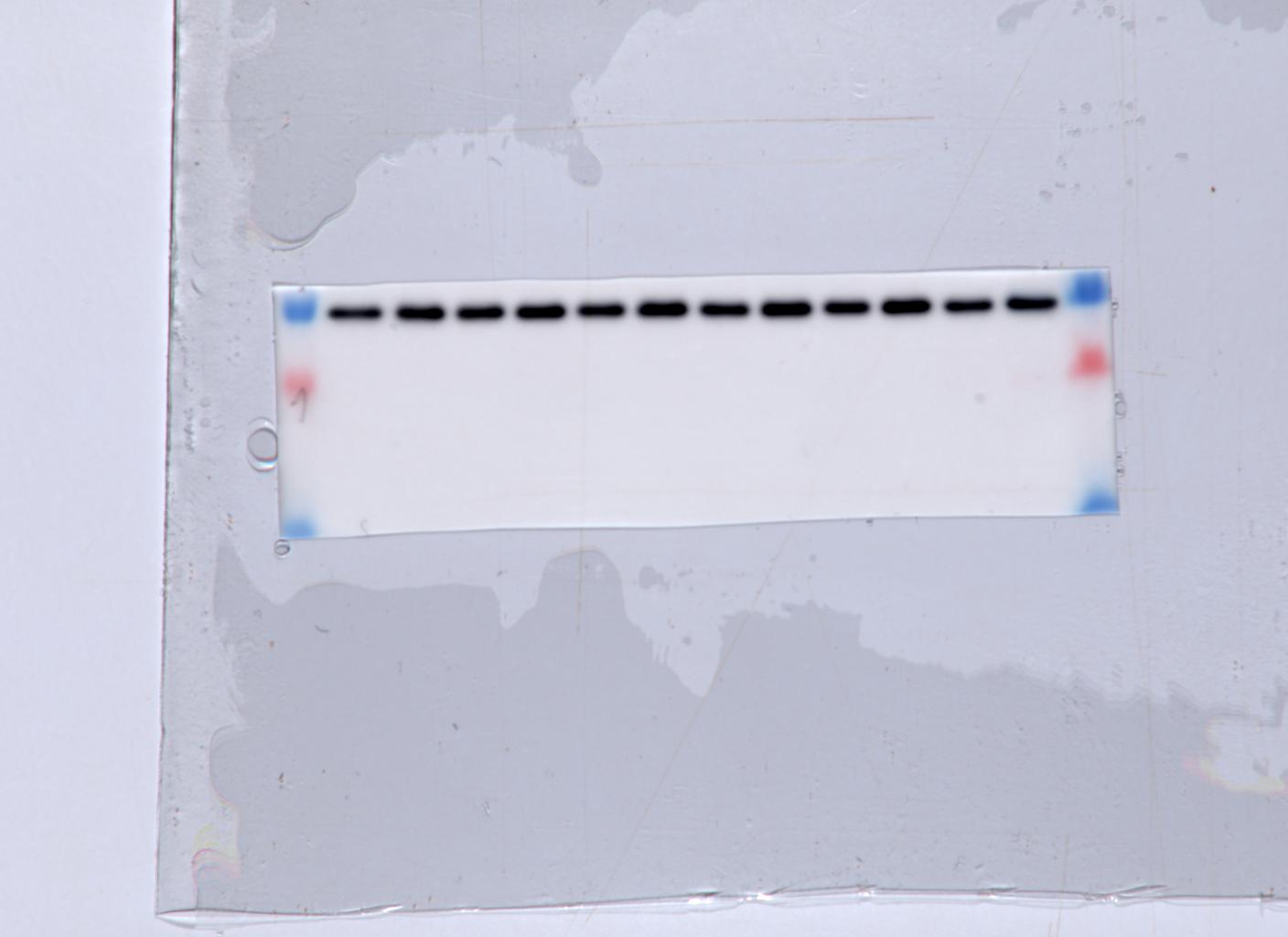

Supplement: Supplementary file 9 — Source data Fig. 2 [file 44321_2024_178_MOESM9_ESM.zip › Figure2 new/2E/Western blot/GAPDH/gel 1 GAPDH 2023.04.13_11.35.13_Ch+Marker.jpg]

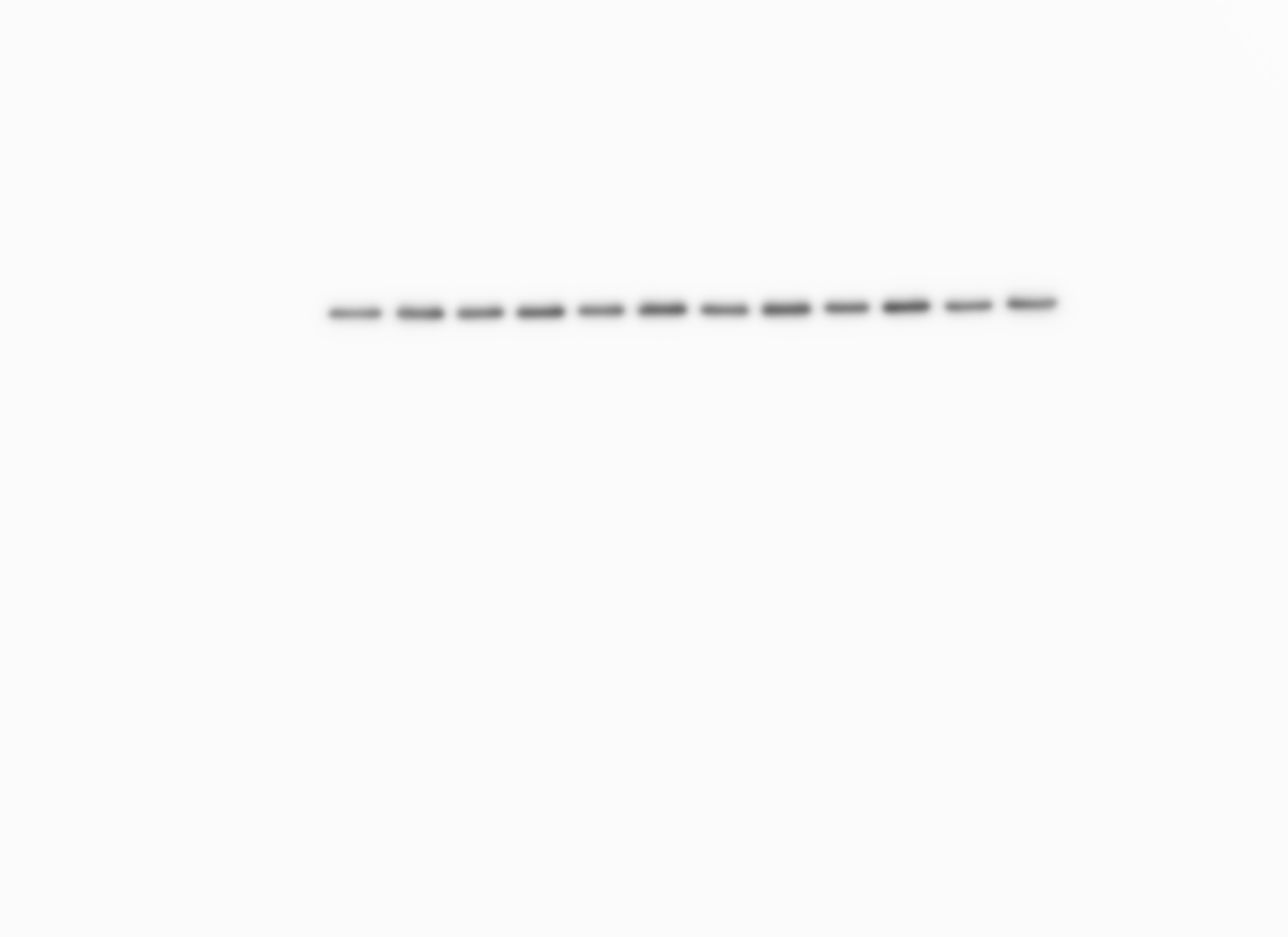

Supplement: Supplementary file 9 — Source data Fig. 2 [file 44321_2024_178_MOESM9_ESM.zip › Figure2 new/2E/Western blot/GAPDH/gel 1 GAPDH 2023.04.13_11.35.13_Ch.tif]

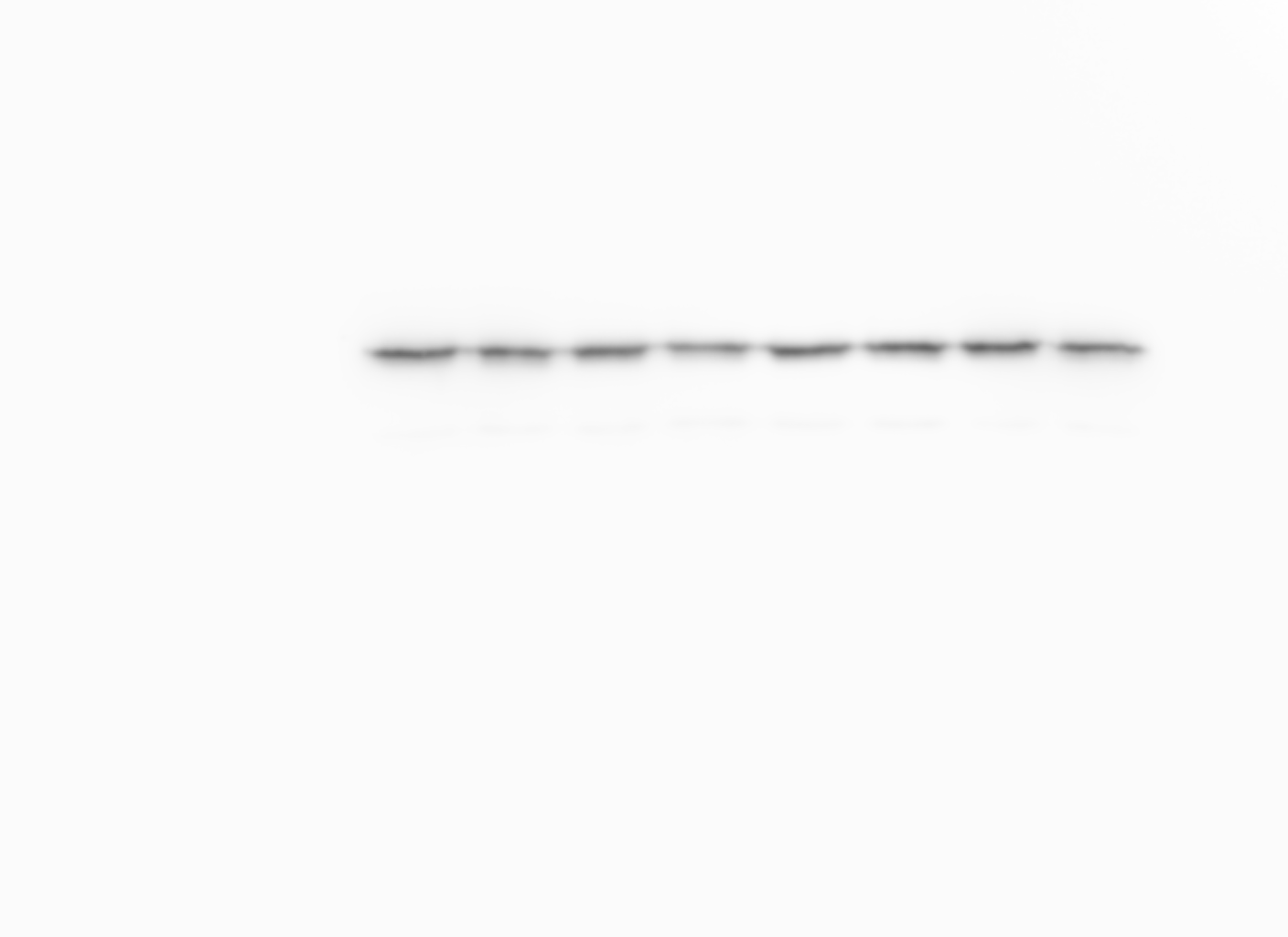

Supplement: Supplementary file 9 — Source data Fig. 2 [file 44321_2024_178_MOESM9_ESM.zip › Figure2 new/2D/Western Blot/GFP/gel4 gfp 2022.12.30_13.01.35_Ch.tif]

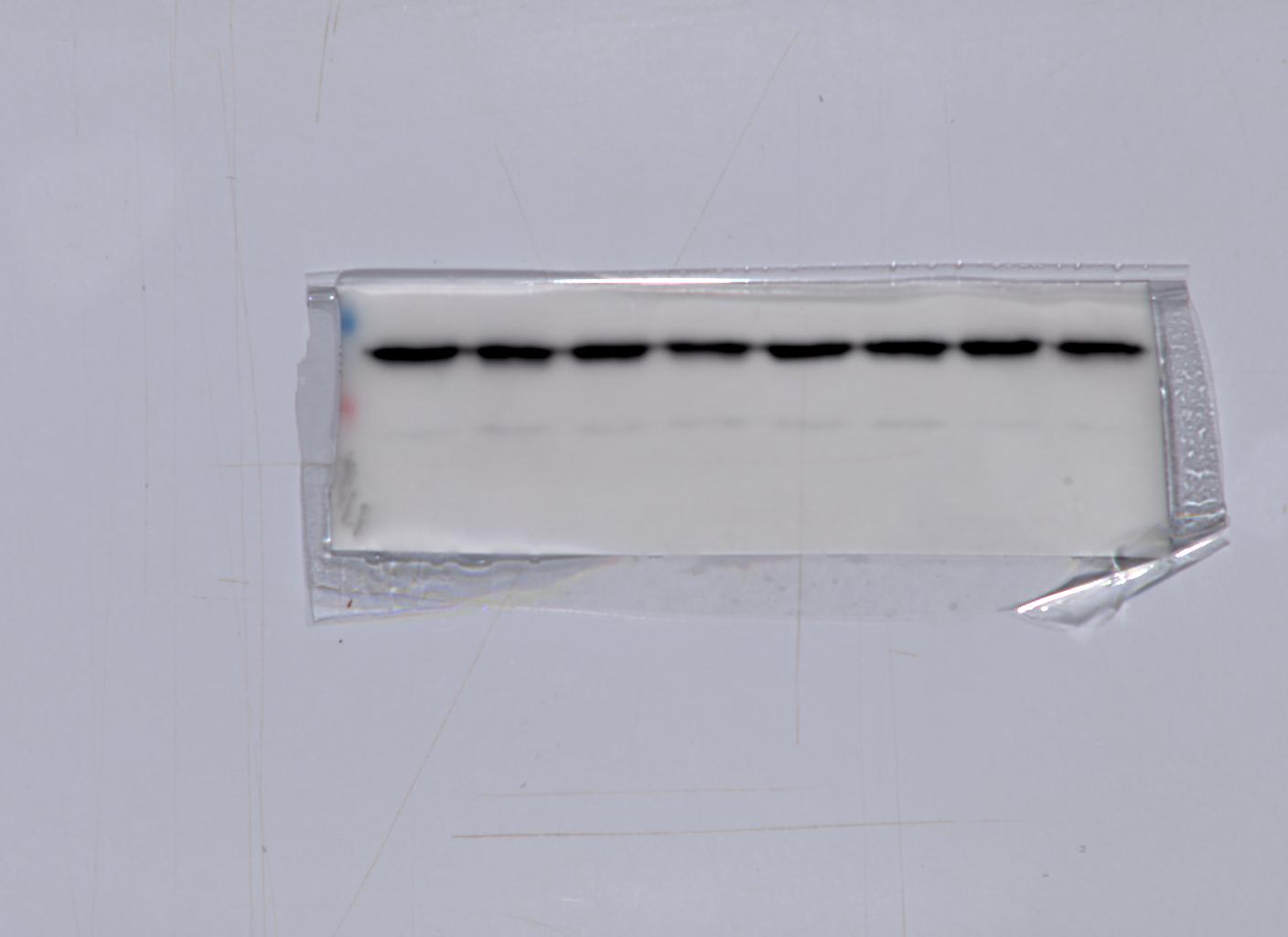

Supplement: Supplementary file 9 — Source data Fig. 2 [file 44321_2024_178_MOESM9_ESM.zip › Figure2 new/2D/Western Blot/GFP/gel4 gfp 2022.12.30_13.01.35_Ch+Marker.jpg]

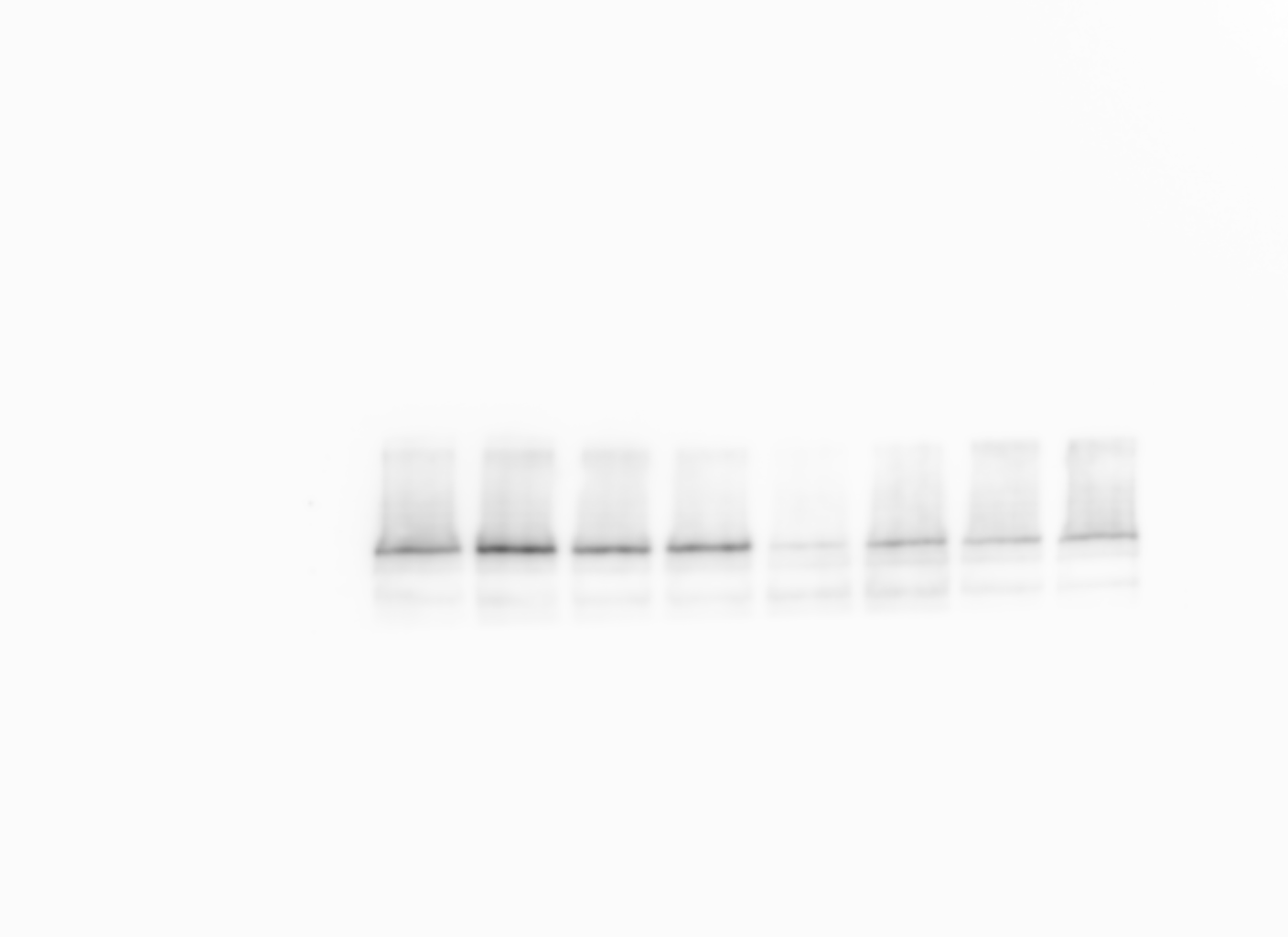

Supplement: Supplementary file 9 — Source data Fig. 2 [file 44321_2024_178_MOESM9_ESM.zip › Figure2 new/2D/Western Blot/HA/gel4 ha 2022.12.30_13.13.13_Ch.tif]

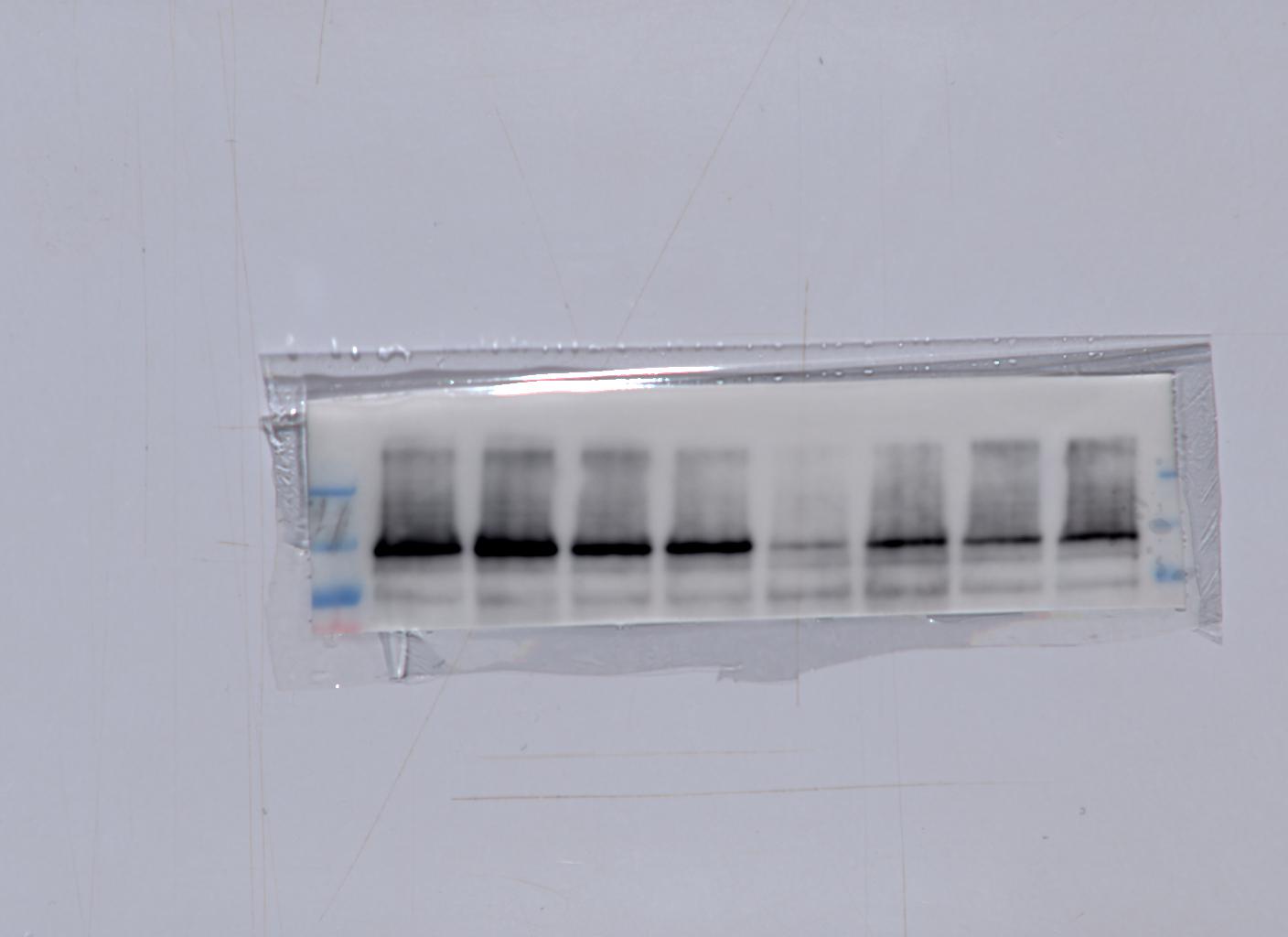

Supplement: Supplementary file 9 — Source data Fig. 2 [file 44321_2024_178_MOESM9_ESM.zip › Figure2 new/2D/Western Blot/HA/gel4 ha 2022.12.30_13.13.13_Ch+Marker.jpg]

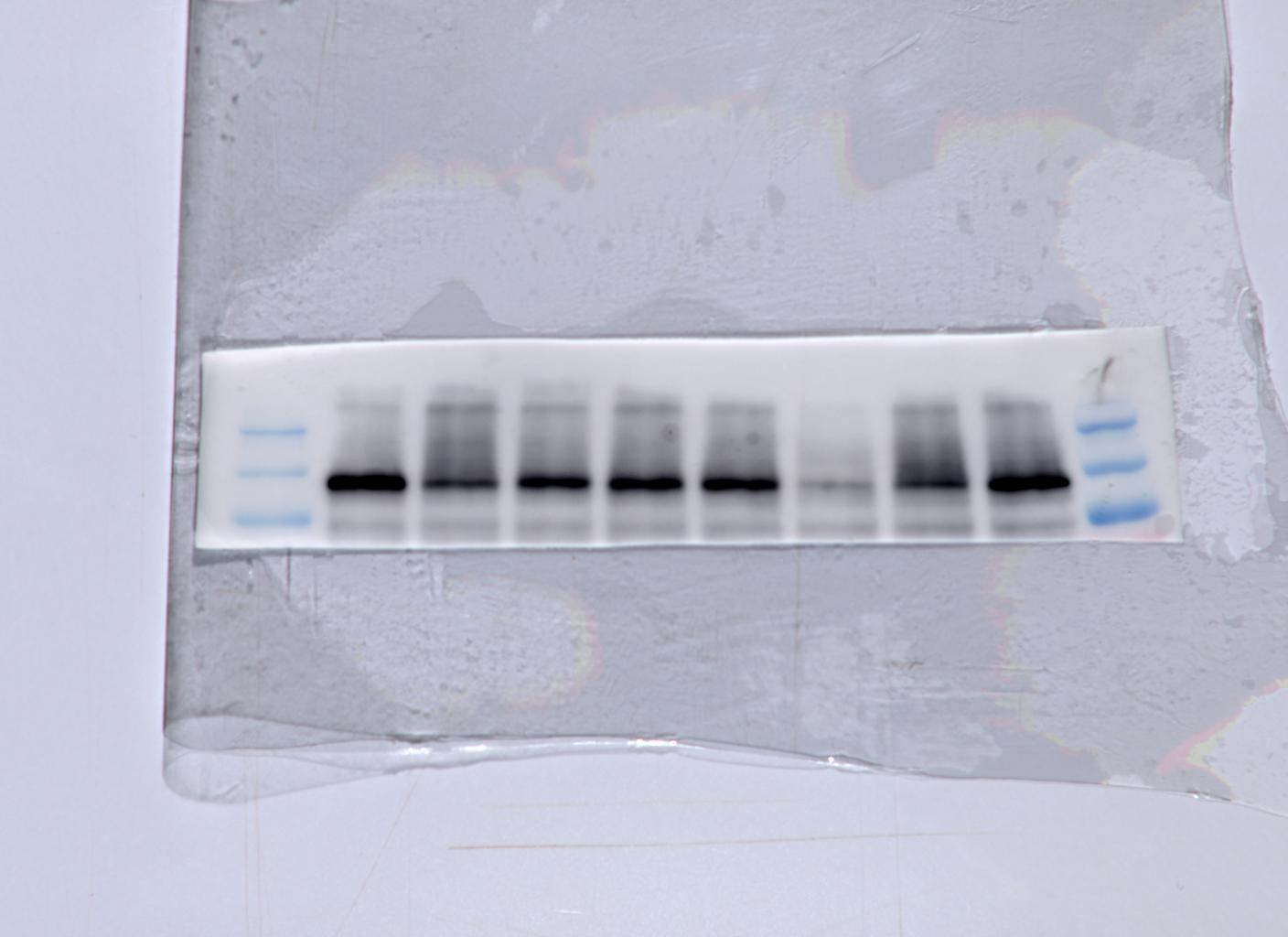

Supplement: Supplementary file 9 — Source data Fig. 2 [file 44321_2024_178_MOESM9_ESM.zip › Figure2 new/2C/Western blot/HA/zwdr47 gel1 fem1 2022.11.30_16.01.51_Ch+Marker.jpg]

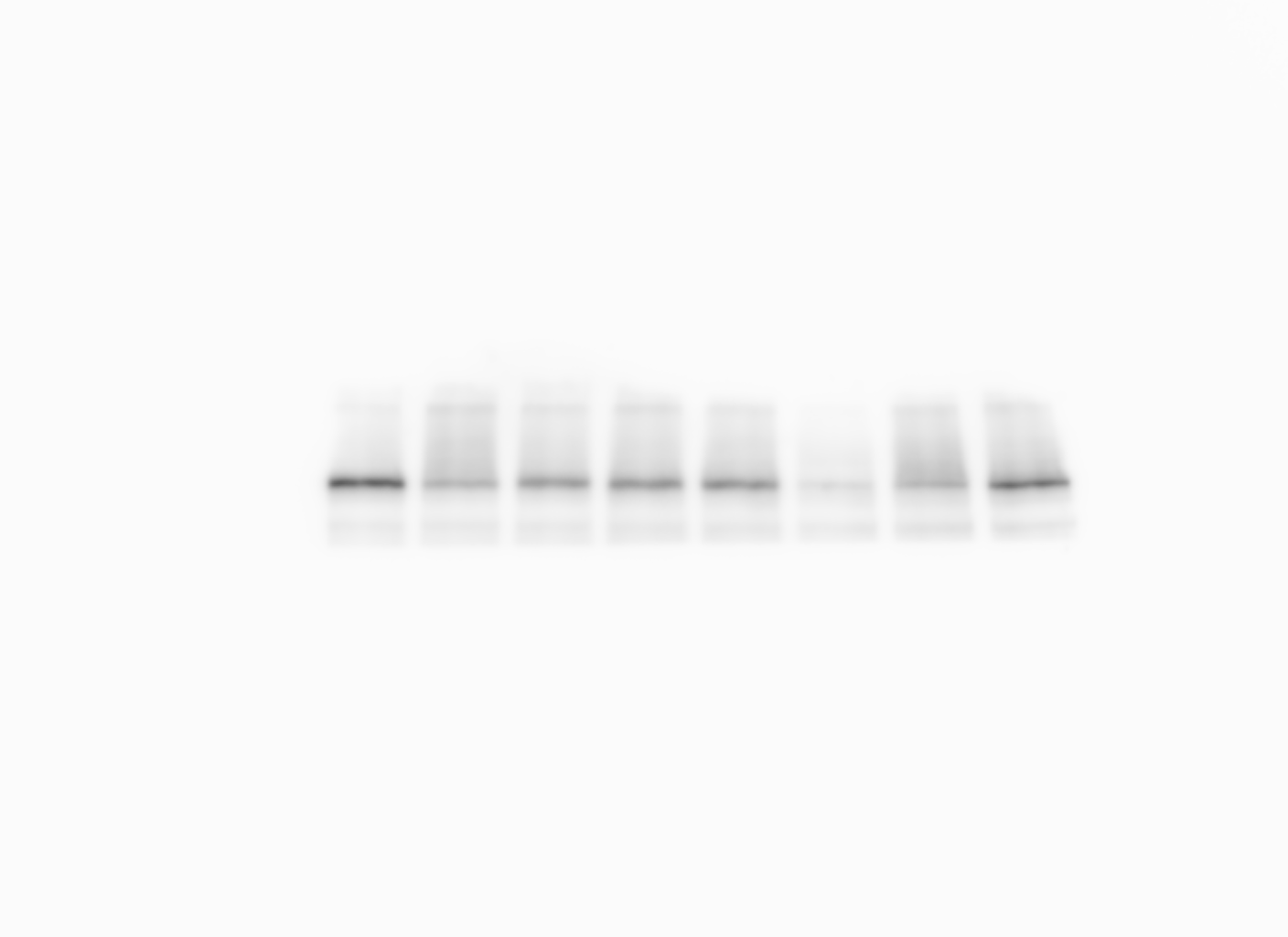

Supplement: Supplementary file 9 — Source data Fig. 2 [file 44321_2024_178_MOESM9_ESM.zip › Figure2 new/2C/Western blot/HA/zwdr47 gel1 fem1 2022.11.30_16.01.51_Ch.tif]

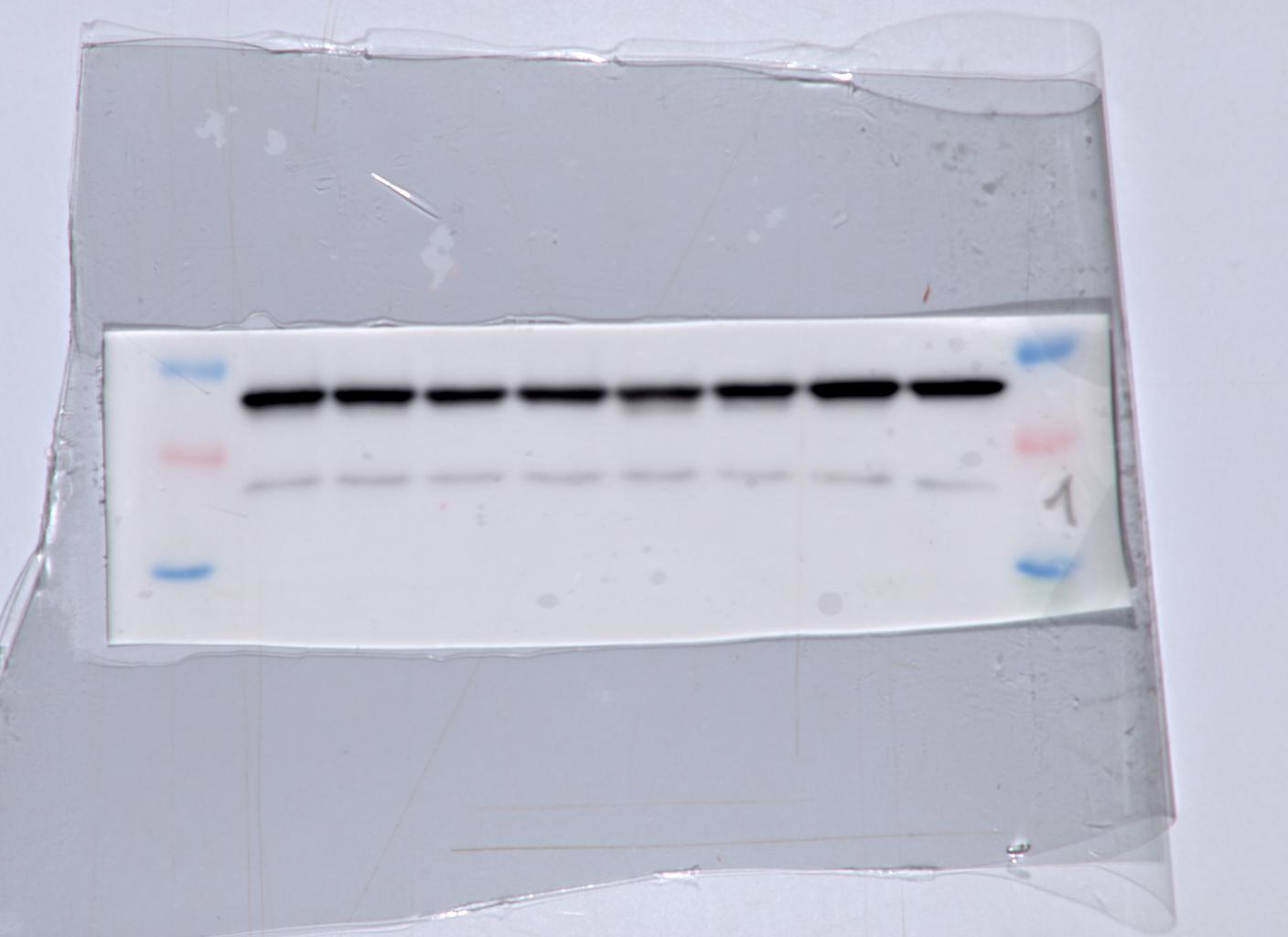

Supplement: Supplementary file 9 — Source data Fig. 2 [file 44321_2024_178_MOESM9_ESM.zip › Figure2 new/2C/Western blot/GFP/gfp gel1 2022.11.30_15.21.26_Ch+Marker.jpg]

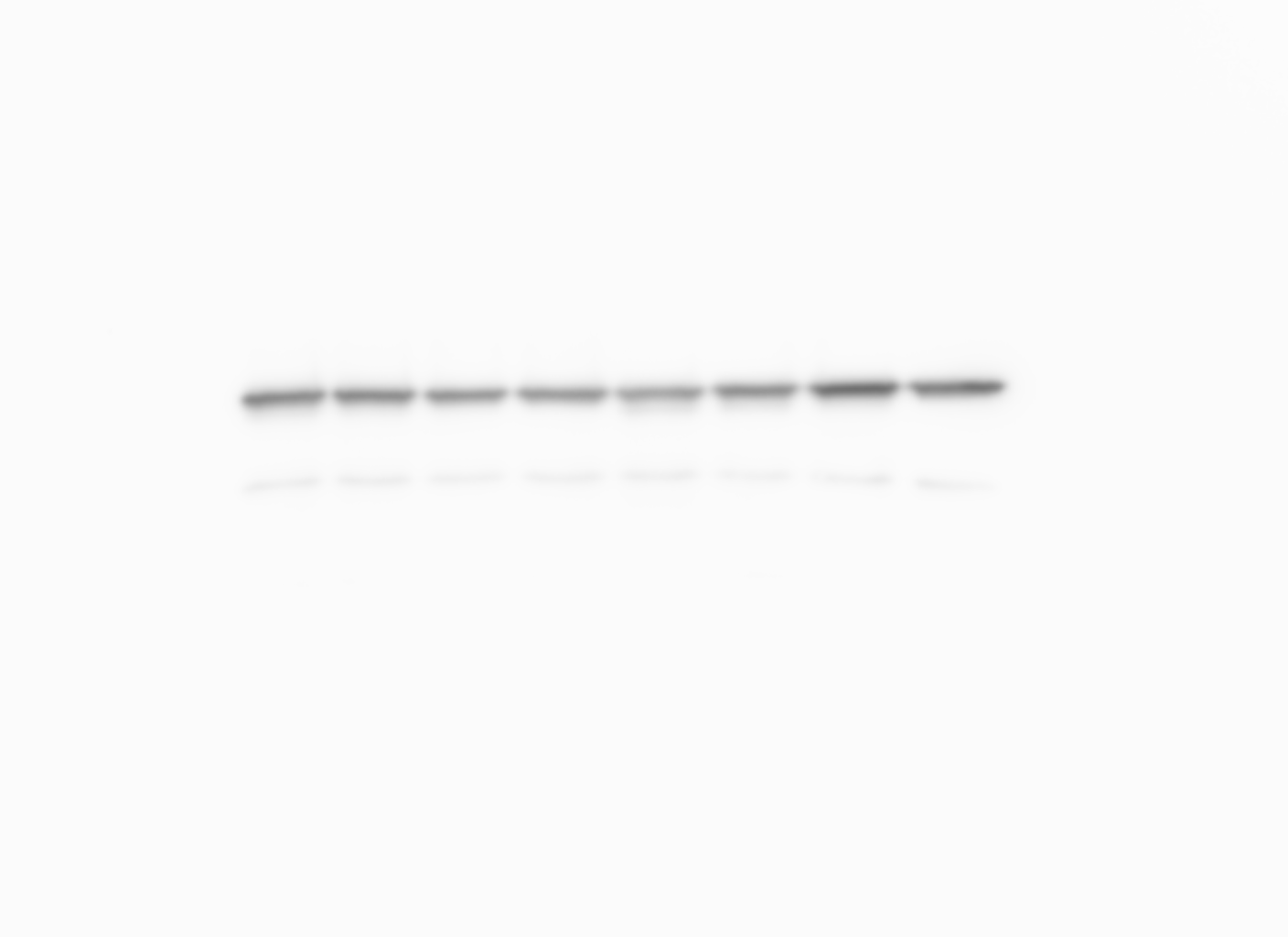

Supplement: Supplementary file 9 — Source data Fig. 2 [file 44321_2024_178_MOESM9_ESM.zip › Figure2 new/2C/Western blot/GFP/gfp gel1 2022.11.30_15.21.26_Ch.tif]

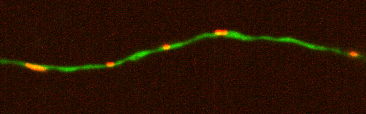

Supplement: Supplementary file 14 — Source data Fig. 7 [file 44321_2024_178_MOESM14_ESM.zip › Fig 7/7E/HOM/HOM MAX 3.17 Composite.tif (RGB).tif]

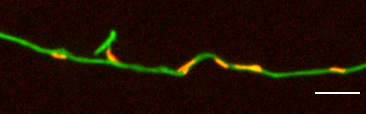

Supplement: Supplementary file 14 — Source data Fig. 7 [file 44321_2024_178_MOESM14_ESM.zip › Fig 7/7E/WT/WTMAX6.27 Composite with scale bar 5 microns.tif]

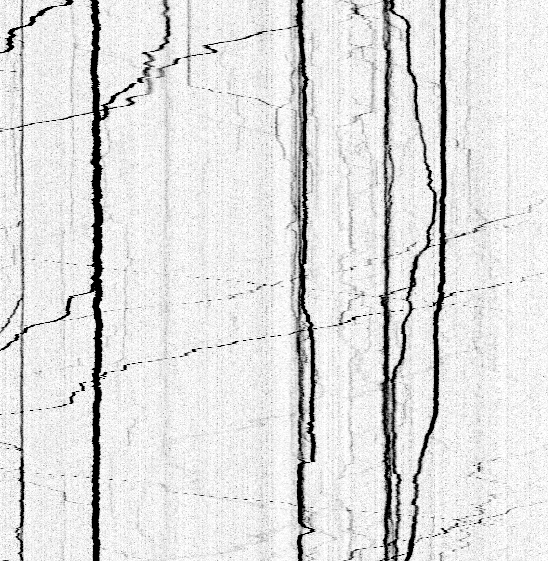

Supplement: Supplementary file 14 — Source data Fig. 7 [file 44321_2024_178_MOESM14_ESM.zip › Fig 7/7L/HOM/Kymograph from C2-Emb 25.2 HOM pic8-1.tif]

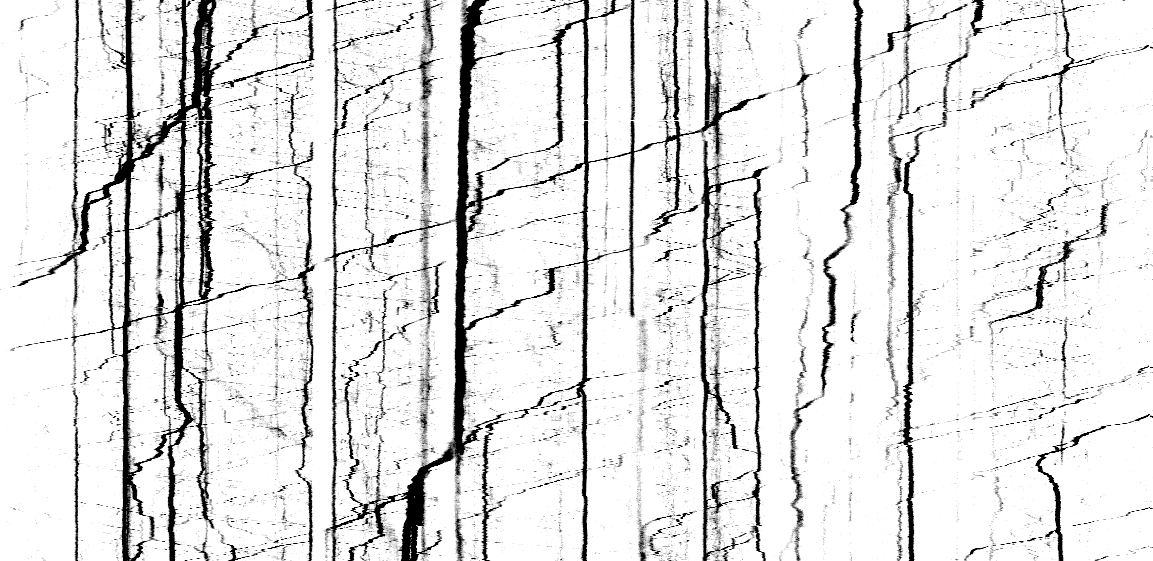

Supplement: Supplementary file 14 — Source data Fig. 7 [file 44321_2024_178_MOESM14_ESM.zip › Fig 7/7L/WT/Kymograph from C2-Emb 25.3 WT pic6-1.tif]

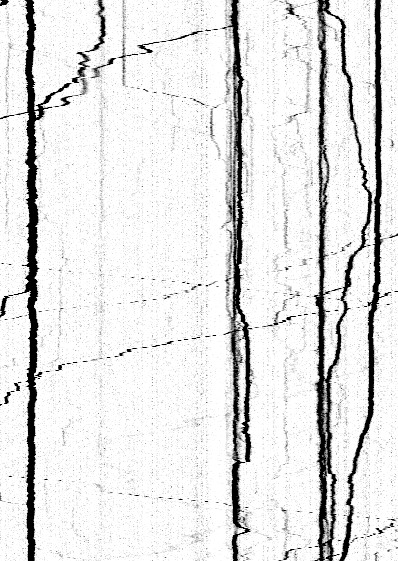

Supplement: Supplementary file 14 — Source data Fig. 7 [file 44321_2024_178_MOESM14_ESM.zip › Fig 7/7L/HOM/cropped 100um/Kymograph from C2-Emb 25.2 HOM pic8-1-1 cut.tif]

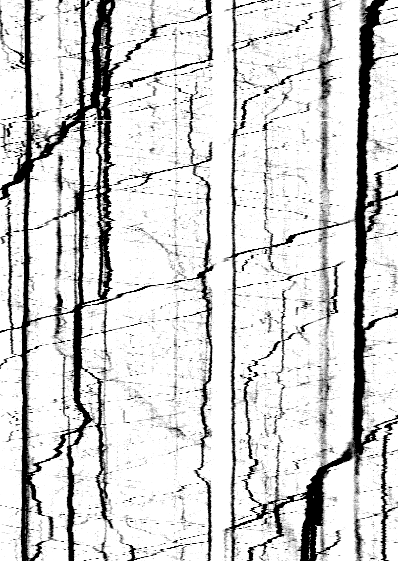

Supplement: Supplementary file 14 — Source data Fig. 7 [file 44321_2024_178_MOESM14_ESM.zip › Fig 7/7L/WT/cropped 100 um/Kymograph from C2-Emb 25.3 WT pic6-1-1 cut.tif]

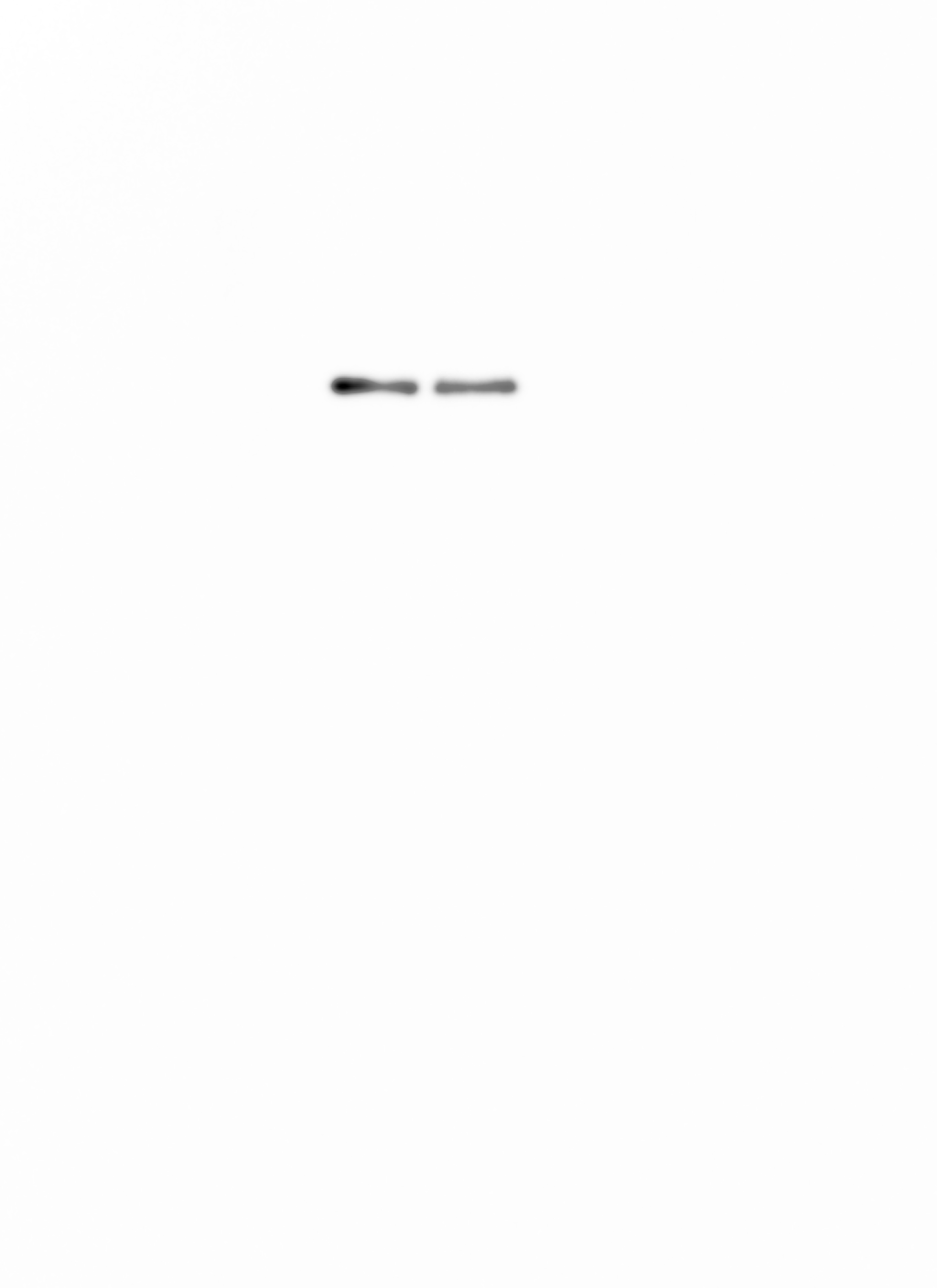

Supplement: Supplementary file 14 — Source data Fig. 7 [file 44321_2024_178_MOESM14_ESM.zip › Fig 7/7N/Western blot/alpha tubulin/alphatubdiv6 1.21m 2022.03.08_14.59.22_Ch.tif]

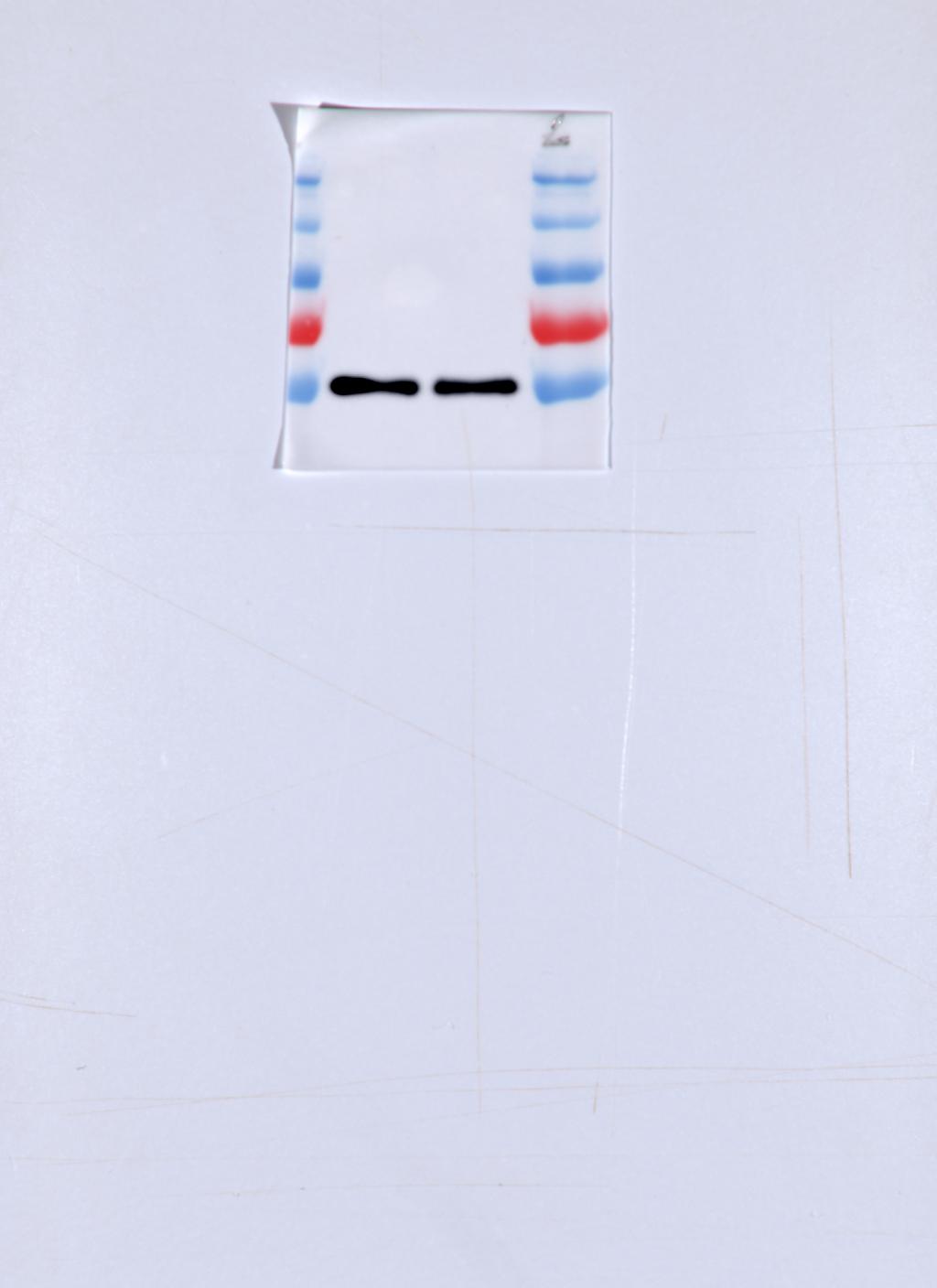

Supplement: Supplementary file 14 — Source data Fig. 7 [file 44321_2024_178_MOESM14_ESM.zip › Fig 7/7N/Western blot/alpha tubulin/alphatubdiv6 1.21m 2022.03.08_14.59.22_Ch+Marker.jpg]

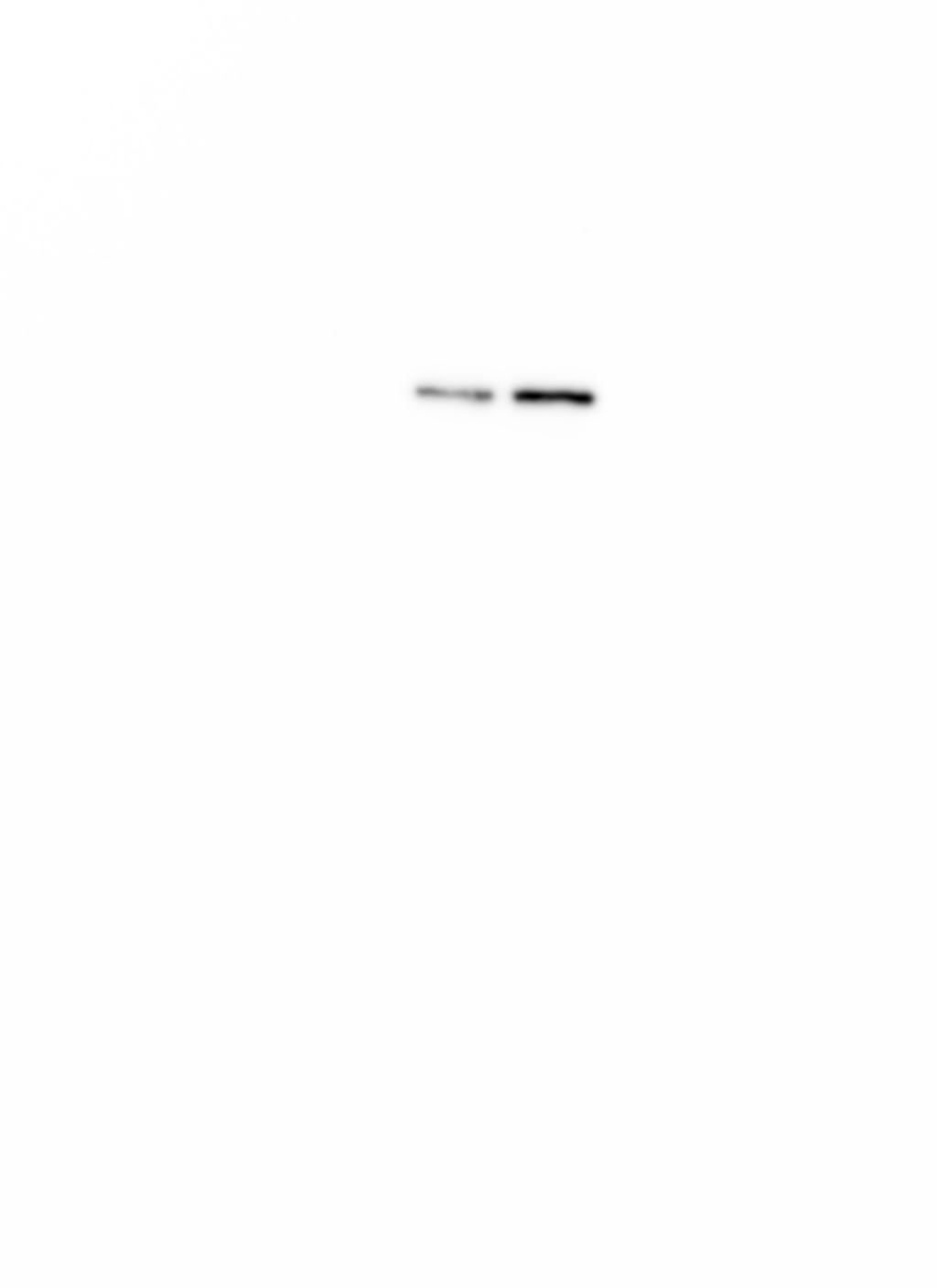

Supplement: Supplementary file 14 — Source data Fig. 7 [file 44321_2024_178_MOESM14_ESM.zip › Fig 7/7N/Western blot/acetylated tubulin/acetyltubdiv6 1.10m 2022.03.08_16.20.27_Ch.tif]

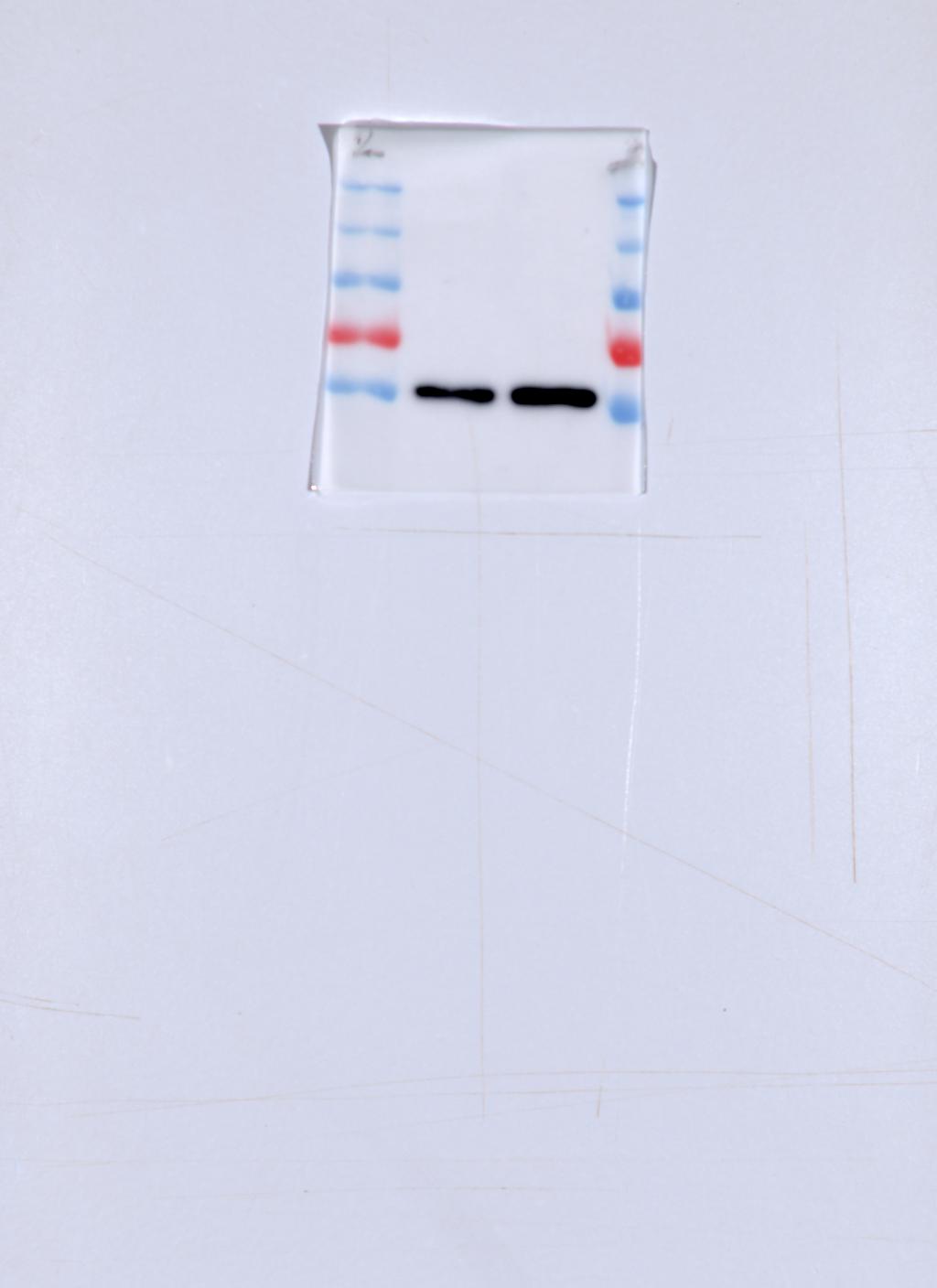

Supplement: Supplementary file 14 — Source data Fig. 7 [file 44321_2024_178_MOESM14_ESM.zip › Fig 7/7N/Western blot/acetylated tubulin/acetyltubdiv6 1.10m 2022.03.08_16.20.27_Ch+Marker.jpg]
