# Supplementary material for: Bi-allelic variants in WDR47 cause a complex neurodevelopmental syndrome
Source: EMBO Mol Med. 2024 Nov 28;17(1):129–68. doi: 10.1038/s44321-024-00178-z (PMC11730659; doi:10.1038/s44321-024-00178-z)
Supplement: Supplementary file 14 — Source data Fig. 7 [file 44321_2024_178_MOESM14_ESM.zip › Fig 7/7N/Western blot/Figure 7N with annotations.pptx]

## Slide 1
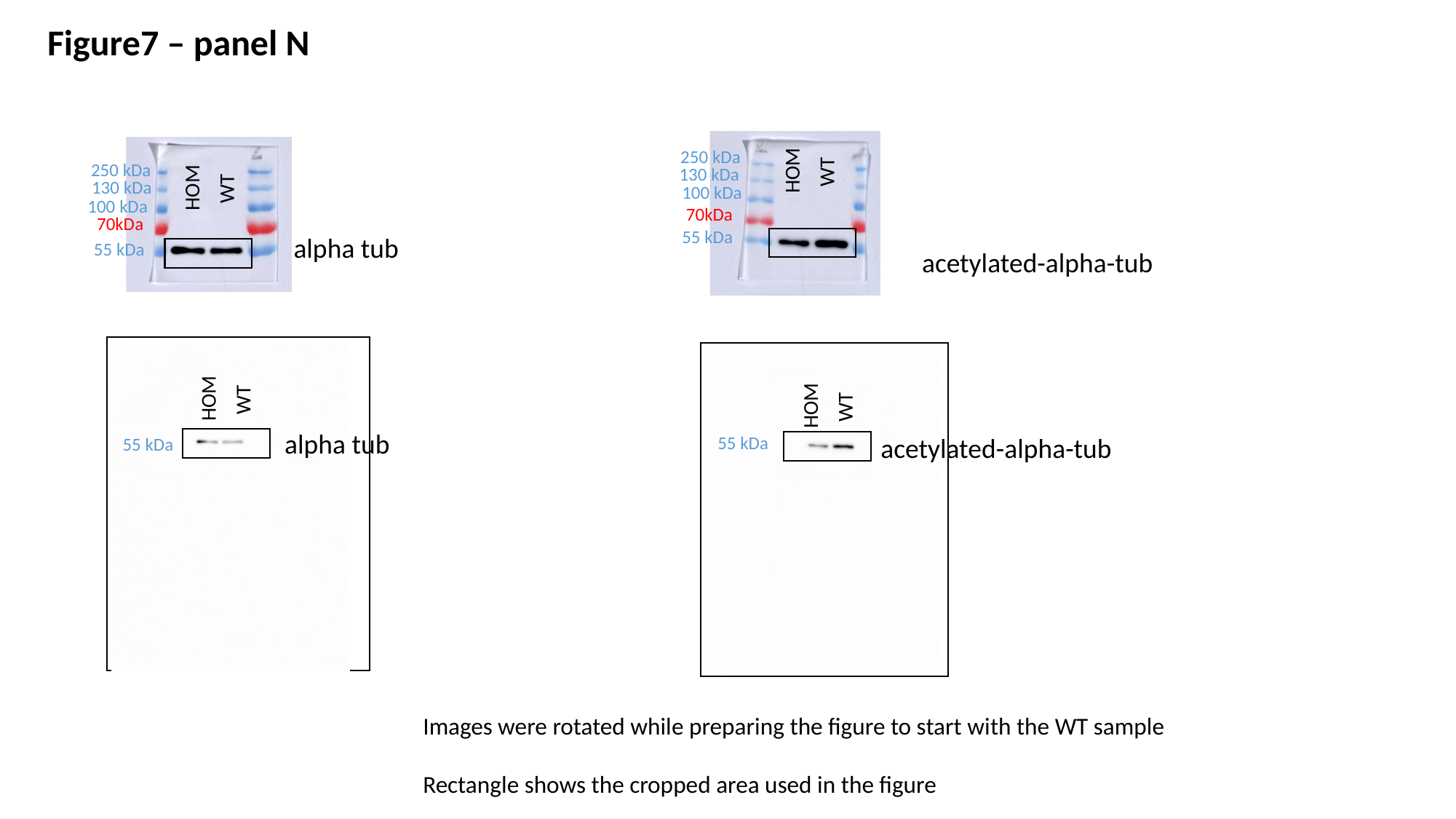

Figure7 – panel N
250 kDa
HOM
250 kDa
WT
130 kDa
HOM
130 kDa
WT
100 kDa
100 kDa
70kDa
70kDa
55 kDa
alpha tub
55 kDa
acetylated-alpha-tub
HOM
WT
HOM
WT
alpha tub
acetylated-alpha-tub
55 kDa
55 kDa
Images were rotated while preparing the figure to start with the WT sample
Rectangle shows the cropped area used in the figure
