# Supplementary material for: Bi-allelic variants in WDR47 cause a complex neurodevelopmental syndrome
Source: EMBO Mol Med. 2024 Nov 28;17(1):129–68. doi: 10.1038/s44321-024-00178-z (PMC11730659; doi:10.1038/s44321-024-00178-z)
Supplement: Supplementary file 16 — Expanded View Figures [file 44321_2024_178_MOESM16_ESM.pdf]

Expanded View Figures

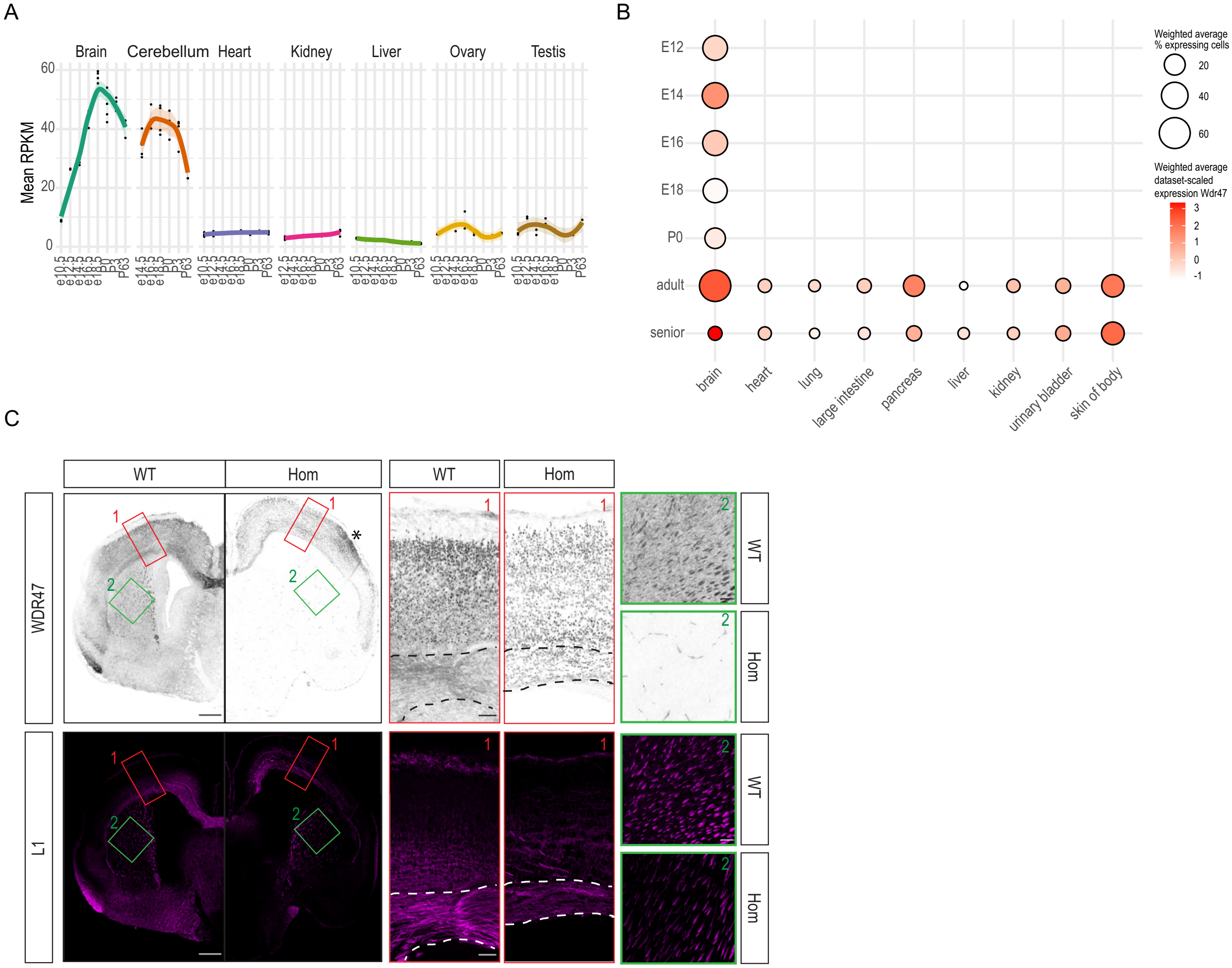

Figure EV1. Pattern of expression of mouse *Wdr47*.

(**A**) Expression values of *Wdr47* as RPKM (Reads per kilo base per million mapped reads) throughout life for different organs in mouse. Dots represent average values for each replicate. Shaded regions represent standard deviation of distribution. Data from (Cardoso-Moreira et al, 2019). (**B**) Expression pattern of *Wdr47* in mice across organs (x axis) and ages (y axis) showing prominent expression in the brain. Data obtained by multiple published dataset through https://cellxgene.cziscience.com (Abdulla et al, 2023). Color scale represents the weighted average expression across datasets and dot size represents the weighted average percentage of cells expressing *Wdr47*. (**C**) Coronal sections of E18.5 WT and HOM (*Wdr47*$^{tm1b/tm1b}$) mouse brains immunostained with WDR47 and L1 antibodies show expression of WDR47 in the cortex with enrichment in fiber tracks. Asterisk depicted unspecific staining. Scale bars: 360 µm and 60 µm (insets).

© The Author(s)

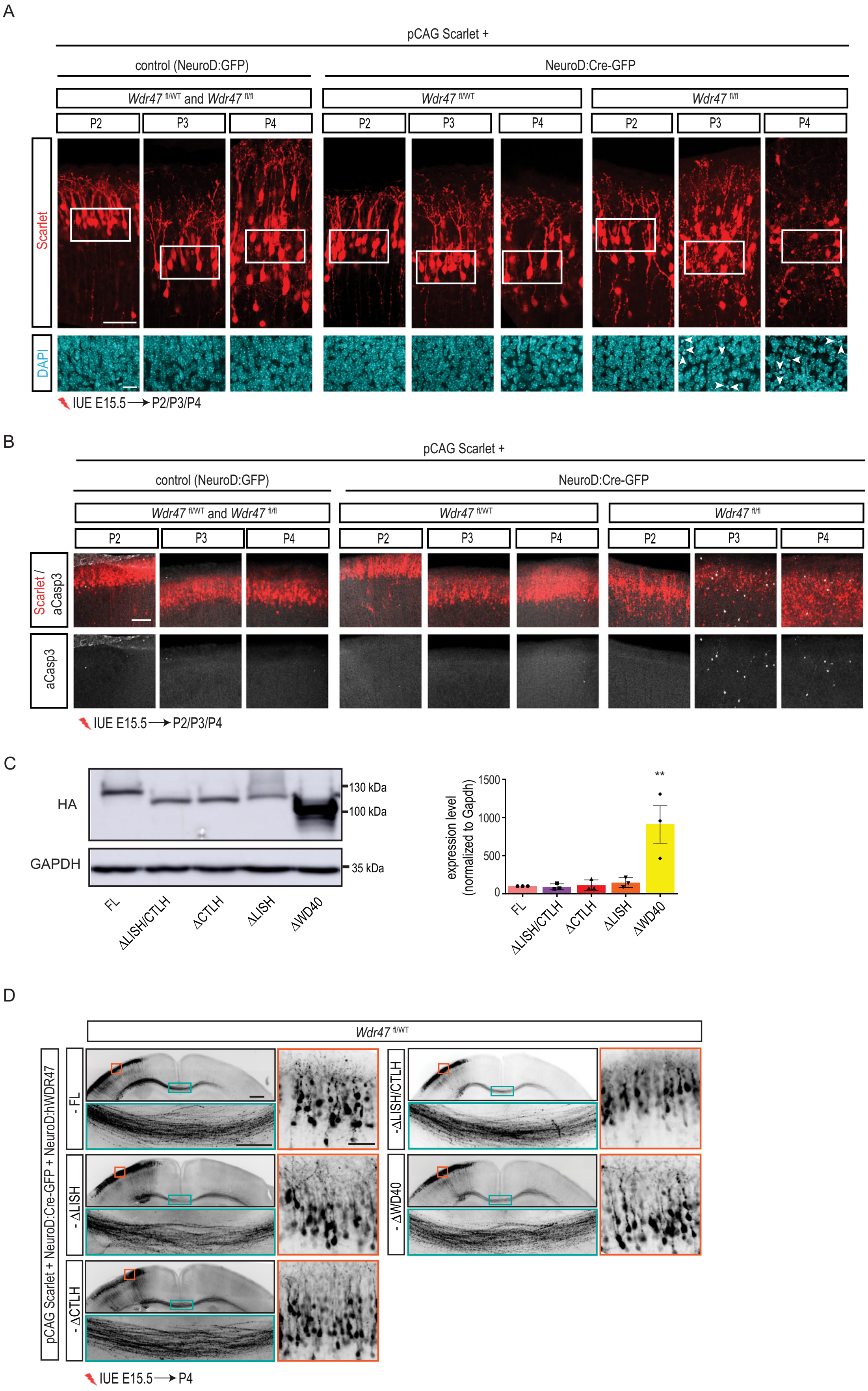
A
pCAG Scarlet +
control (NeuroD:GFP)
NeuroD:Cre-GFP
Wdr47 fl/WT and Wdr47 fl/fl
Wdr47 fl/WT
Wdr47 fl/fl
P2
P3
P4
Scarlet
DAPI
IUE E15.5 → P2/P3/P4
B
pCAG Scarlet +
control (NeuroD:GFP)
NeuroD:Cre-GFP
Wdr47 fl/WT and Wdr47 fl/fl
Wdr47 fl/WT
Wdr47 fl/fl
P2
P3
P4
Scarlet / aCasp3
aCasp3
IUE E15.5 → P2/P3/P4
C
HA
GAPDH
130 kDa
100 kDa
35 kDa
FL
ΔLISH/CTLH
ΔCTLH
ΔLISH
ΔWD40
expression level (normalized to Gapdh)
1500
1000
500
0
**
D
Wdr47 fl/WT
pCAG Scarlet + NeuroD:Cre-GFP + NeuroD:hWDR47
- FL
- ΔLISH/CTLH
- ΔLISH
- ΔWD40
- ΔCTLH
IUE E15.5 → P4

Figure EV2. Loss of *Wdr47* induces massive neuronal death at early postnatal stages.

(**A**, **B**) Coronal sections of P2, P3, and P4 mouse brains electroporated at E15.5 with pCAG:Scarlet plasmid together with either a control (NeuroD:Ires:GFP) or a NeuroD:Cre-GFP vector. Scarlet positive electroporated neurons are depicted in red. (**A**) While *Wdr47*$^{fl/WT}$ neurons and neurons in control conditions keep a proper morphology, *Wdr47*$^{fl/fl}$ neurons lose their bipolar morphology from P3 on. In close-up views of the white boxed area, nucleus is counterstained with DAPI and arrowheads point to pyknotic nuclei. Scale bars: 50 µm and 20 µm (insets). (**B**) Coronal sections are immunolabelled for activated Caspase3 (aCasp3) (white). Several aCasp3+ cells appear at P3 and P4 in *Wdr47*$^{fl/fl}$ condition. Scale bar: 100 µm. (**C**) Western blot analysis of extracts from HEK cells transfected with the indicated HA tagged hWDR47 truncated constructs. Gapdh is used as the loading control. Data (means ± s.d.) from at least 3 independent experiments were analyzed by one-way ANOVA, with Bonferroni's multiple comparisons test. $^{**}P < 0.01$. Note that ΔWD40 construct is expressed about 10 times more than the other constructs. (**D**) Effect of different rescue constructs on CC and neuronal survival in control conditions. Coronal sections of P4 *Wdr47*$^{fl/WT}$ mouse brains electroporated at E15.5 with pCAG:Scarlet and NeuroD:Cre-GFP plasmids together with a truncated WDR47 construct. Scarlet positive electroporated neurons are depicted in black. Close-up views of the green and orange boxed area show no effect of the construct on the CC and neuronal survival. Data from at least 3 independent experiments. Scale bars: 500 µm, 200 µm (green boxed inset) and 50 µm (red boxed inset). Exact *P* values are listed in Dataset EV4.

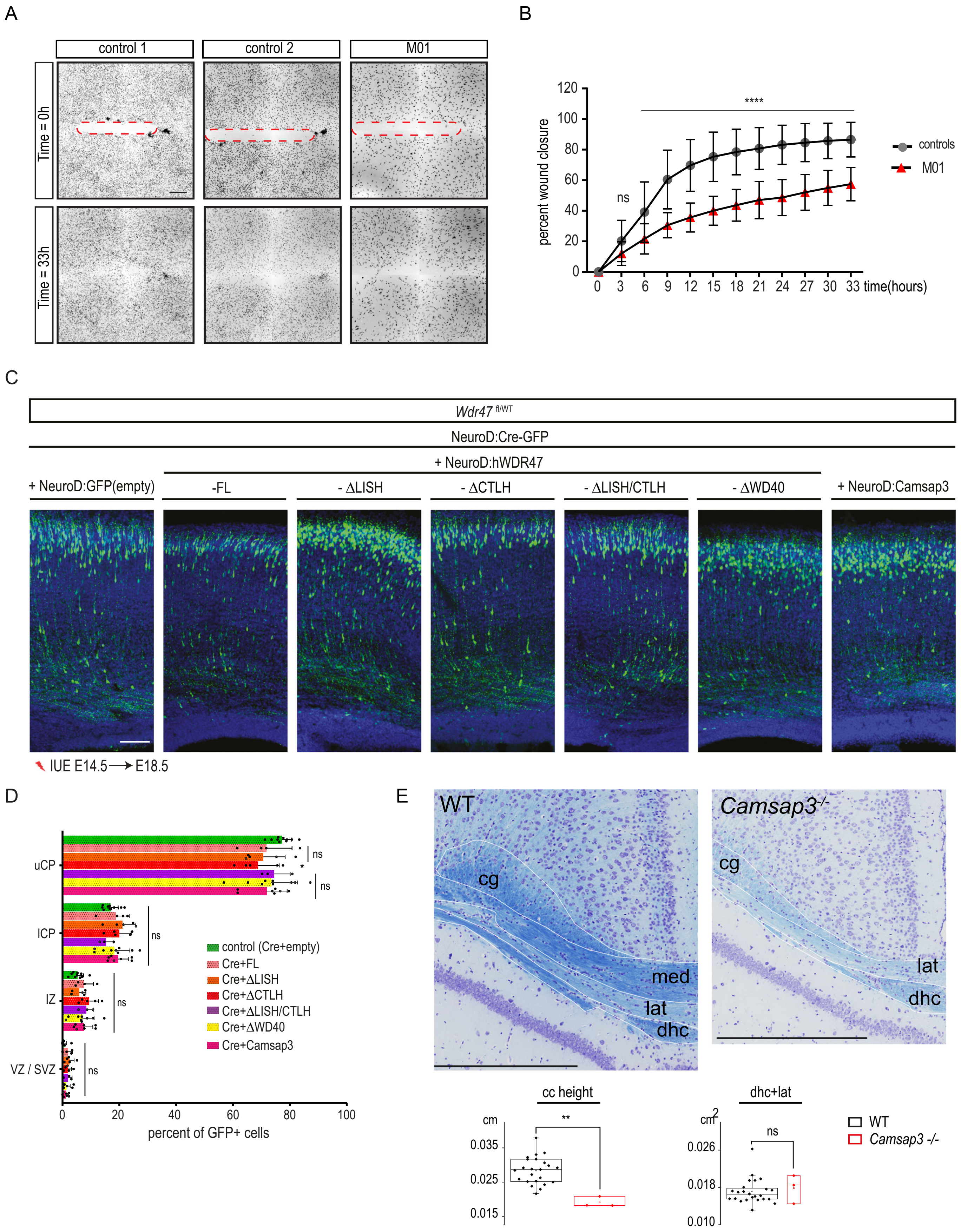
A
control 1
control 2
M01
Time = 0h
Time = 33h
B
percent wound closure
ns
****
controls
M01
time(hours)
C
Wdr47 fl/WT
NeuroD:Cre-GFP
+ NeuroD:hWDR47
+ NeuroD:GFP(empty)
-FL
- ΔLISH
- ΔCTLH
- ΔLISH/CTLH
- ΔWD40
+ NeuroD:Camsap3
IUE E14.5 → E18.5
D
uCP
lCP
IZ
VZ / SVZ
percent of GFP+ cells
control (Cre+empty)
Cre+FL
Cre+ΔLISH
Cre+ΔCTLH
Cre+ΔLISH/CTLH
Cre+ΔWD40
Cre+Camsap3
E
WT
Camsap3-/-
cg
med
lat
dhc
cc height
dhc+lat
WT
Camsap3 -/-

◀ **Figure EV3. Effect of different rescue constructs on neuronal migration in control conditions.**

(**A**) Transmission light micrographs of an in vitro 36-h neuronal migration assay performed on Mitomycin-treated fibroblast lines obtained from two healthy subjects and patient M01. The dashed red lines show the edge of the wound. Scale bar: 500 µm. (**B**) Percentage of wound closure is shown over time in M01-derived fibroblasts compared to control lines. Data (means ± s.d.) from at least 18 wells per condition was analyzed by two-way ANOVA, with Bonferroni's multiple comparison test, ns, non-significant, $^{****}P < 0.0001$. (**C**) Coronal sections of E18.5 *Wdr47*$^{fl/WT}$ mouse cortices 4 days after in utero electroporation with NeuroD-Cre-GFP together with a different NeuroD construct used for rescue experiments. GFP-positive electroporated cells are depicted in green. Nuclei are stained with DAPI. The representative image of NeuroD:GFP (empty) electroporation in *Wdr47*$^{fl/WT}$ embryo is identical to the one shown in Fig. 5A. Scale bar: 100 µm. (**D**) Analysis of the percentage of electroporated GFP-cells in different regions (uCP, lCP, IZ, and VZ/SVZ) show that apart from the mild effect of ΔWD40 domain construct, none of the constructs have an effect on neuronal migration in control conditions. Data (means ± s.d.) from at least three embryos per condition were analyzed by two-way ANOVA, with Bonferroni's multiple comparisons test, ns, non-significant; $^{*}P < 0.05$. uCP, Upper cortical plate; lCP, Lower cortical plate; IZ, intermediate zone; VZ, ventricular zone; SVZ, subventricular zone. (**E**) Top: Representative brain image stained with Nissl-luxol of adult male WT and *Camsap3* KO mice showing the soma of the corpus callosum at Bregma −1.34 mm. Bottom: Box plot showing the combined size of the lateral fibers (lat) of the corpus callosum with the hippocampal commissure in 3 male *Camsap3*$^{-/-}$ and 24 matched baseline WT mice of 16 weeks of age bred on a pure genetic background C57BL/6N. The line in the middle represents the median, the upper limit of the box corresponds to Q3, the lower limit to Q1, and the whiskers extend to 1.5× interquartile range. Scale bar: 0.05 cm. Data (means ± s.d.) was analyzed by two-tailed Student's *t*-tests of equal variances. ns, non-significant; $^{**}P < 0.01$. cg, cingulate bundle; dhc, dorsal hippocampal commissure; lat, lateral fibers; med, medial fibers. Exact *P* values are listed in Dataset EV4.

© The Author(s)

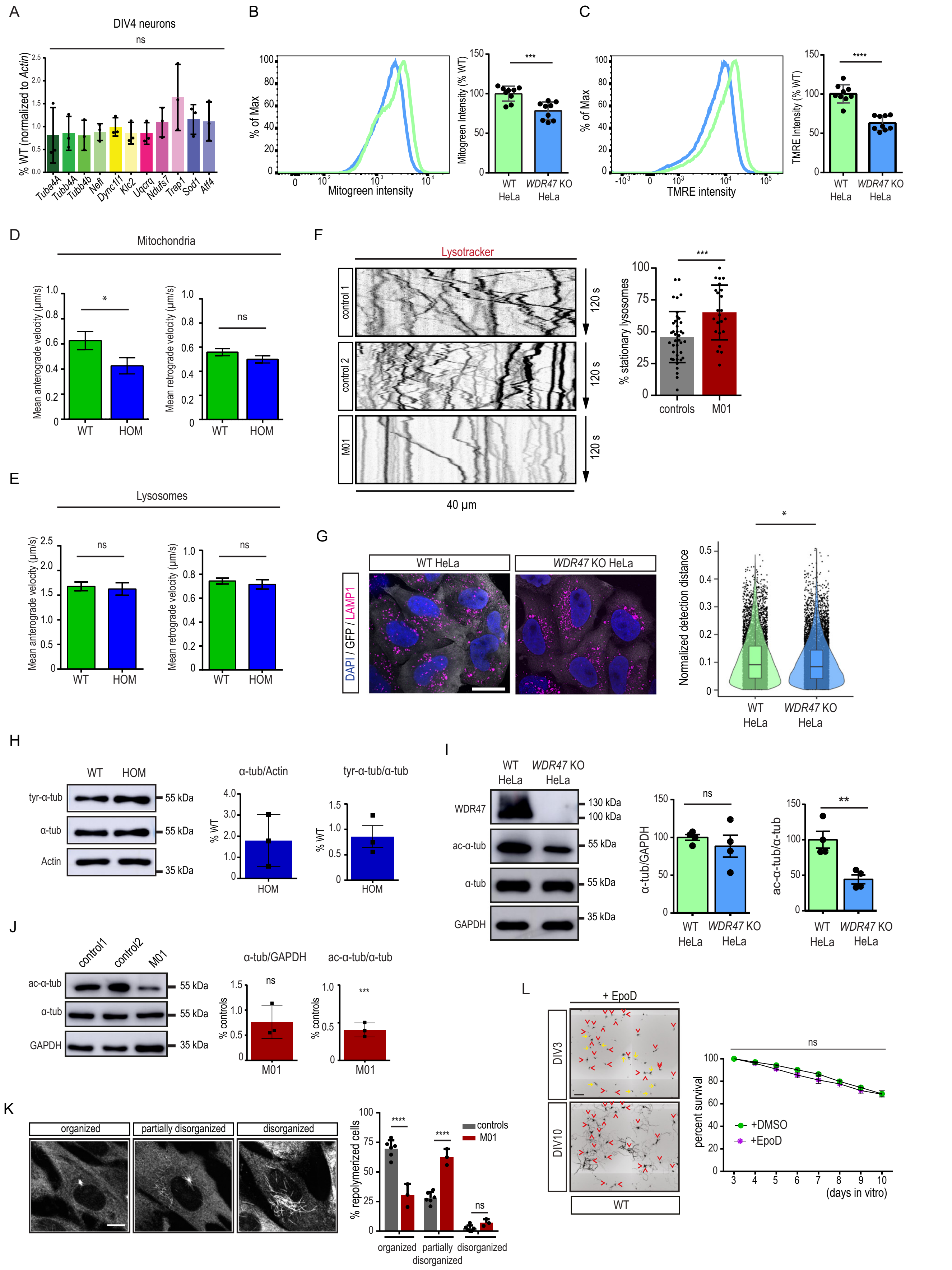
A
DIV4 neurons
ns
% WT (normalized to Actin)
Tuba4A
Tubb4A
Tubb4b
Nefl
Dync1l1
Klc2
Uqcrq
Ndufs7
Trap1
Sod1
Atf4
B
% of Max
Mitogreen intensity
Mitogreen Intensity (% WT)
***
WT HeLa
WDR47 KO HeLa
C
% of Max
TMRE intensity
TMRE Intensity (% WT)
****
WT HeLa
WDR47 KO HeLa
D
Mitochondria
Mean anterograde velocity (µm/s)
*
WT
HOM
Mean retrograde velocity (µm/s)
ns
WT
HOM
E
Lysosomes
Mean anterograde velocity (µm/s)
ns
WT
HOM
Mean retrograde velocity (µm/s)
ns
WT
HOM
F
Lysotracker
control 1
control 2
M01
120 s
120 s
120 s
40 µm
% stationary lysosomes
***
controls
M01
G
WT HeLa
WDR47 KO HeLa
DAPI / GFP / LAMP1
Normalized detection distance
*
WT HeLa
WDR47 KO HeLa
H
WT
HOM
tyr-α-tub
α-tub
Actin
55 kDa
55 kDa
35 kDa
α-tub/Actin
% WT
HOM
tyr-α-tub/α-tub
% WT
HOM
I
WT HeLa
WDR47 KO HeLa
WDR47
ac-α-tub
α-tub
GAPDH
130 kDa
100 kDa
55 kDa
55 kDa
35 kDa
α-tub/GAPDH
ns
WT HeLa
WDR47 KO HeLa
ac-α-tub/α-tub
**
WT HeLa
WDR47 KO HeLa
J
control1
control2
M01
ac-α-tub
α-tub
GAPDH
55 kDa
55 kDa
35 kDa
α-tub/GAPDH
ns
% controls
M01
ac-α-tub/α-tub
***
% controls
M01
K
organized
partially disorganized
disorganized
% repolymerized cells
****
****
ns
controls
M01
organized
partially disorganized
disorganized
L
+ EpoD
DIV3
DIV10
WT
ns
percent survival
+DMSO
+EpoD
(days in vitro)

Figure EV4. Loss of *Wdr47* in neurons does not impair intracellular transport or pool of dynamic microtubules.

(**A**) RT-qPCR using extracts from DIV4 primary cortical neurons, for the 11 genes that were validated to be differently expressed at DIV6. Data (means ± s.d.) from at least 3 independent cultures per condition was analyzed by unpaired t-test. ns, non-significant. (**B**) Mitochondrial mass and (**C**) mitochondrial membrane potential quantified using flow cytometry in WT and *WDR47* KO HeLa cells. Data (means ± s.d.) from 9 independent experiments was analyzed by unpaired t-test, ***$P < 0.001$, ****$P < 0.0001$. (**D**, **E**) Histograms represent mean anterograde and retrograde velocities of (**D**) mitochondria (anterograde velocity: $n = 77$ for WT and $n = 36$ for HOM; retrograde velocity: $n = 130$ for WT and $n = 88$ for HOM) and (**E**) lysosomes (anterograde velocity: $n = 216$ for WT and $n = 116$ for HOM; retrograde velocity: $n = 408$ for WT and $n = 173$ for HOM). Data (means ± SEM) from at least 3 independent cultures per condition was analyzed by unpaired t-test with Welch correction, ns, non-significant, *$P < 0.05$. (**F**) Kymographs illustrating the motility of lysosomes (Lysotracker) in control and mutant fibroblast in time (y, sec) and space (x, µm). Histograms representing the percentage of stationary lysosomes. Lysosomes from $n = 39$ and $n = 23$ cells were analyzed for control lines and fibroblasts derived from MO1, respectively, and data (means ± s.d.) was analyzed by unpaired t-test, ***$P < 0.001$. (**G**) LAMP1 immunostainings showing increased clustering of lysosomes around the nucleus in *WDR47*-KO HeLa cells compared to WT control HeLa cells. Cells were transfected with GFP to label their cytoplasm. Scale bar: 10 µm. $n = 7138$ and $n = 8447$ lysosomes from 94 WT and *WDR47*-KO HeLa cells was analyzed, respectively, by nested mixed effect model. Box plot: center line: median; box limits: 1st and 3rd quartiles; whiskers: $+/-1.5\times$ interquartile range. *$P < 0.05$. (**H**) Western blot analysis showing unchanged levels of alpha tubulin (α-tub) and tyrosinated alpha tubulin (tyr-α-tub) in DIV6 HOM ($Wdr47^{tm1b/tm1b}$) primary neurons compared to WT. Actin was used as loading control. Data (means ± s.d.) from 3 independent cultures was analyzed by unpaired t-test. (**I**, **J**) Western blot analysis showing decreased levels of acetylated tubulin (ac-α-tub) and unchanged levels of alpha tubulin (α-tub) in (**I**) *WDR47* KO HeLa cells compared to WT HeLa cells and (**J**) fibroblasts derived from M01 compared to fibroblasts derived from healthy individuals. GAPDH was used as loading control. Data (means ± s.d.) from 3 to 4 independent cultures was analyzed by unpaired t-test, ns, non-significant, **$P < 0.01$; ***$P < 0.001$. (**K**) Representative images of human primary fibroblasts categorized as organized, partially disorganized and disorganized depending on the nucleation pattern of microtubules after 30 min of depolymerization followed by 2 min of repolymerization. Percentage of cells in each category is shown in M01-derived fibroblasts compared to control lines. >50 cells were analyzed for each culture and data (means ± s.d.) from 3 cultures was analyzed by two-way ANOVA, with Bonferroni's multiple comparison test, ns, non-significant; ****$P < 0.0001$. (**L**) Effect of EpoD on WT cultures. (Right) Representative fields, at DIV3 and DIV10, of WT neuronal cultures treated with EpoD at DIV2. Scarlet positive electroporated neurons are depicted in black. Yellow arrows correspond to neurons that died, red arrowheads correspond to neurons that are alive and could be followed from DIV3 to DIV10. (Left) Survival of WT neurons from DIV3 to DIV10 upon treatment with DMSO and EpoD. Data (means ± s.d.) from at least 3 cultures per condition was analyzed by two-way ANOVA, with Bonferroni's multiple comparison test. ns, non-significant. Exact *P* values are listed in Dataset EV4.

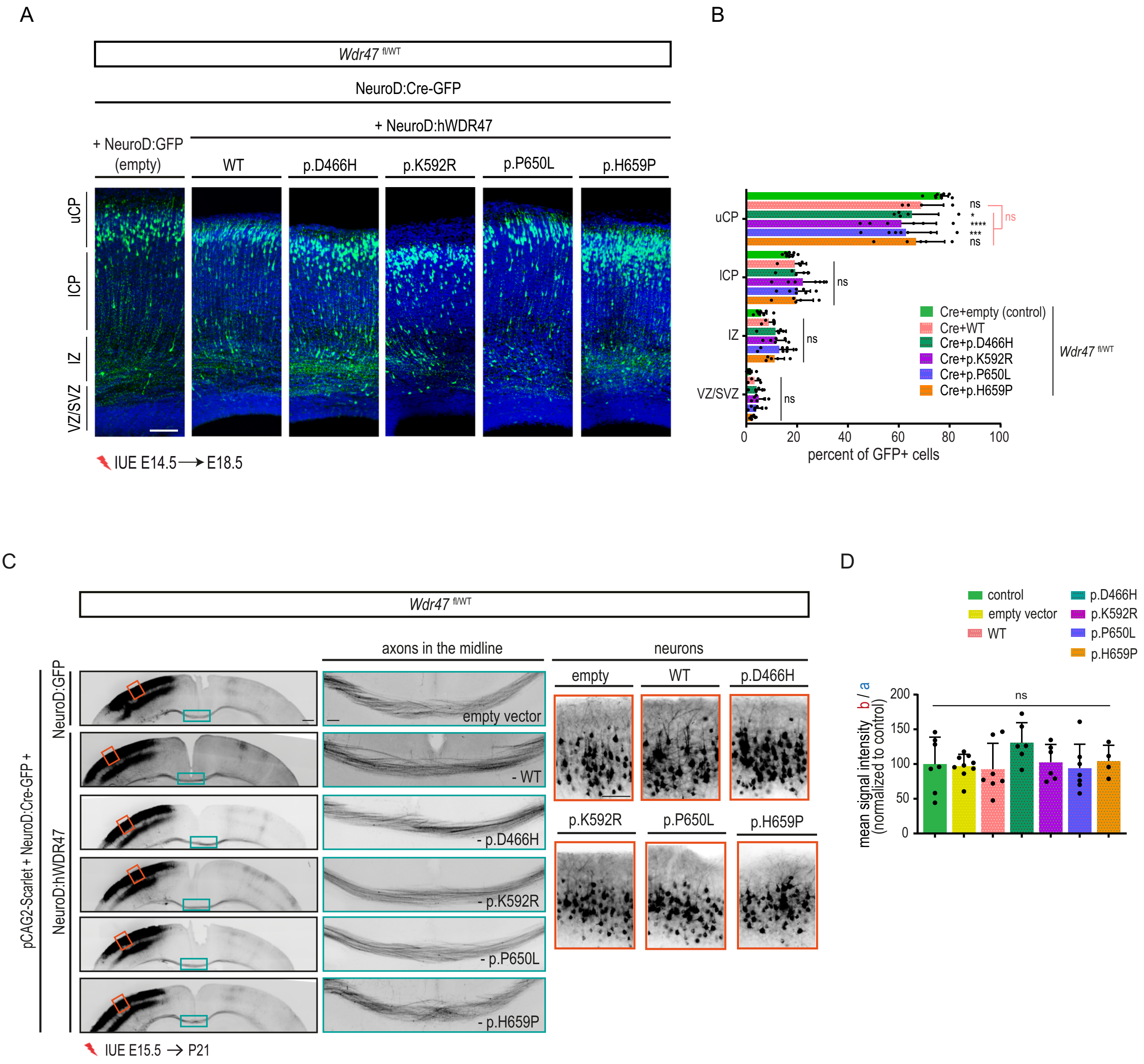

Figure EV5. Effect of wild-type and mutant hWDR47 constructs on CC and neuronal survival in control conditions.

(**A**) Coronal sections of E18.5 *Wdr47*$^{fl/WT}$ mouse cortices, 4 days after in utero electroporation with NeuroD-Cre-GFP together with an empty (NeuroD:GFP) or wild type (WT) or mutant NeuroD:WDR47 constructs. GFP-positive electroporated cells are depicted in green. Nuclei are stained with DAPI. Scale bar: 100 μm. (**B**) Analysis of the percentage of electroporated GFP-cells in different regions (uCP, lCP, IZ, and VZ/SVZ) showing a mild effect of overexpression of some mutant WDR47 in the upper cortical plate (uCP). Data (means ± s.d.) from at least five embryos from 2 to 4 different litters per condition was analyzed by two-way ANOVA, with Bonferroni's multiple comparisons test. uCP, Upper cortical plate; lCP, Lower cortical plate; IZ, intermediate zone; VZ, ventricular zone; SVZ, subventricular zone. (**C**) Coronal sections of P21 *Wdr47*$^{fl/WT}$ mouse brains electroporated at E15.5 with pCAG:Scarlet and NeuroD:Cre-GFP plasmids and together with either an empty (NeuroD:GFP) or a NeuroD:WDR47 construct with or without the human mutation. Scarlet positive electroporated neurons are depicted in black. Close-up views of the green boxed and red boxed areas show that none of the constructs have an effect on CC or neuronal morphology. Scale bars: 500 μm, 100 μm (green and red boxed insets). (**D**) CC thickness upon introduction of different hWDR47 constructs. Data (means ± s.d.) from at least 5 pups per condition were analyzed by one-way ANOVA, with Bonferroni's multiple comparison test. ns, non-significant; *$P < 0.05$; ***$P < 0.001$; ****$P < 0.0001$. Exact P values are listed in Dataset EV4.

© The Author(s)
